# Targeting MTAP increases PARP inhibitor susceptibility in triple-negative breast cancer through a feed-forward loop

**DOI:** 10.1172/JCI188120

**Published:** 2025-07-01

**Authors:** Xiangyu Zeng, Fei Zhao, Xinyi Tu, Yong Zhang, Wen Yang, Jing Hou, Qi Jiang, Shouhai Zhu, Zheming Wu, Yalan Hao, Lingxin Zhang, Richard M. Weinshilboum, Kaixiong Tao, Liewei Wang, Zhenkun Lou

**Affiliations:** 1 Department of Gastrointestinal Surgery, Union Hospital, Tongji Medical College, Huazhong University of Science and Technology, Wuhan, China.; 2 College of Biology, Hunan University, Changsha, China.; 3 Department of Oncology, Mayo Clinic, Rochester, Minnesota, USA.; 4 Department of Radiation Oncology, Hubei Cancer Hospital, Tongji Medical College, and; 5 Department of Breast and Thyroid Surgery, Union Hospital, Tongji Medical College, Huazhong University of Science and Technology, Wuhan, China.; 6 Department of Breast Surgery, Guizhou Provincial People’s Hospital, Guiyang, China.; 7 Analytical Instrumentation Center, Hunan University, Changsha, China.; 8 Department of Molecular Pharmacology and Experimental Therapeutics, Mayo Clinic, Rochester, Minnesota, USA.

**Keywords:** Oncology, Therapeutics, Amino acid metabolism, Breast cancer, DNA repair

## Abstract

Triple-negative breast cancer (TNBC) represents the most malignant subtype of breast cancer. The clinical application of PARP inhibitors (PARPi) is limited by the low frequency of *BRCA1/2* mutations in TNBC. Here, we identified that *MTAP* deletion sensitized genotoxic agents in our clinical cohort of metastatic TNBC. Further study demonstrated that *MTAP* deficiency or inhibition rendered TNBC susceptibility to chemotherapeutic agents, particularly PARPi. Mechanistically, targeting MTAP that synergized with PARPi by disrupting the METTL16-MAT2A axis involved in methionine metabolism and depleting in vivo s-adenosylmethionine (SAM) levels. Exhausted SAM in turn impaired PARPi-induced DNA damage repair through attenuation of MRE11 recruitment and end resection by diminishing MRE11 methylation. Notably, brain metastatic TNBC markedly benefited from a lower dose of PARPi and *MTAP* deficiency/inhibition synergy due to the inherently limited methionine environment in the brain. Collectively, our findings revealed a feed-forward loop between methionine metabolism and DNA repair through SAM, highlighting a therapeutic strategy of PARPi combined with *MTAP* deficiency/inhibition for TNBC.

## Abstract 2

Targeting MTAP confers TNBC to PARP inhibitors

## Graphical abstract

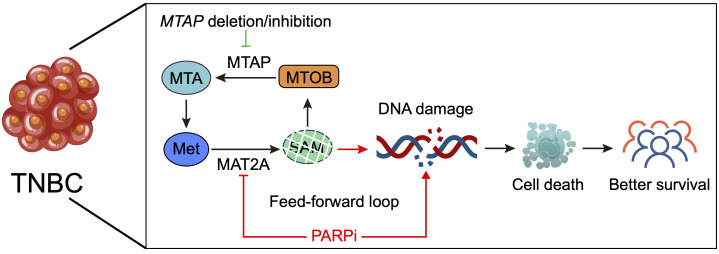

## Introduction

Breast cancer is the most frequently diagnosed cancer among women worldwide (1). Although triple-negative breast cancer (TNBC) constitutes approximately 15% of all breast cancer cases, it accounts for nearly 25% of breast cancer–related deaths (2, 3). The development of targeted therapies for TNBC is challenging because of its molecular heterogeneity and the absence of therapeutic target (4, 5). Surgical intervention and chemotherapy remain the mainstay treatments for most patients with TNBC. While early-stage TNBC generally shows favorable responses to treatment, advanced-stage disease is associated with a substantially higher likelihood of relapse and metastasis compared with other subtypes (6). Metastatic TNBC presents a particularly dismal prognosis, with over 50% of patients with metastasis succumbing within 1 year of diagnosis, especially in cases involving brain metastasis (BrM) (7, 8). Therefore, there is a critical and unmet need to develop more effective therapeutics for TNBC.

Methylthioadenosine (MTA) phosphorylase (MTAP) is a key metabolic enzyme involved in the methionine (Met) salvage pathway that converts MTA to Met (9). *MTAP* is often homozygously deleted as a consequence of a passenger event, in which neighbor gene cyclin-dependent kinase inhibitor 2A (*CDKN2A*) is deleted, with a frequency of approximately 15% in cases of pan-cancer and exceeding 50% in certain cancer types (10, 11). Mounting evidence demonstrated that *MTAP* deficiency represents a synthetically targetable vulnerability in conjunction with MAT2A or PRMT5 (9–15). Nonetheless, clinical application of MAT2A or PRMT5 inhibitors is hindered by the low efficacy and cellular adaptations.

Poly (ADP-ribose) polymerase (PARP) inhibitors (PARPi) have emerged as a promising therapeutic strategy for cancers with germline variants of *BRCA*1/2 by leading to the accumulation of DNA double-strand breaks (DSBs) (16, 17). PARPi has come to the forefront of breast cancer treatment in recent years. In 2018, olaparib and talazoparib were approved for the treatment of germline *BRCA*-mutated metastatic breast cancer, based on their remarkable improvement of progression-free survival in 2 phase III randomized clinical trials (18, 19). However, it is noteworthy that only about 10% of patients with breast cancer harbor germline or somatic *BRCA1* and *BRCA2* mutations (20, 21), leaving most patients missing out on the encouraging treatment strategy. Besides deleterious germline *BRCA* variants, other molecular alterations within tumors may also lead to similar defects in homologous recombination (HR) (22–24). This phenomenon, often referred to as “BRCAness,” suggests the potential to expand the eligible patient population for PARPi therapy. Various molecular characteristics underpinning BRCAness have been elucidated (22–24); however, the mechanisms by which PARPi may exert effects beyond DNA repair and gene alteration in additional pathways as potential synthetic targets remain to be fully investigated.

Here, we identified that *MTAP* deletion confers vulnerability to genotoxic agents in our metastatic TNBC cohort study. Subsequent cellular and mouse studies indicated that PARPi synergized markedly with *MTAP* deficiency/inhibition by disrupting the METTL16-MAT2A axis in Met metabolism. The resultant SAM depletion initiated a feed-forward loop with PARPi-induced DNA damage, leading to diminished MRE11 recruitment and end resection. Met restriction (MR) enhanced the combination efficacy of PARPi and *MTAP* deficiency/inhibition, and therefore a lower dose of PARPi can exhibit pronounced synergistic effect with *MTAP* deficiency or inhibition in brain metastatic TNBC, attributed by the intrinsically restricted Met availability in the brain. Collectively, these findings proposed a promising therapeutic strategy for TNBC, highlighting the feed-forward interaction between Met metabolism and DNA repair mechanisms.

## Results

### MTAP deletion or inhibition shows susceptibility to PARPi.

During the course of their disease, more than one-third of patients with TNBC will develop distant metastases (25, 26), which represents a severe threat to their overall survival. Treatment for these patients predominantly relies on systemic chemotherapy. However, the median duration of response to first-line chemotherapy ranges from 4.4 to 6.6 months, while a second-line or subsequent therapies yield a duration of 4.2 to 5.9 months (2, 3). Improved understanding the heterogeneity of this disease and identification of potential biomarkers for response are urgently needed.

**Figure 1:**
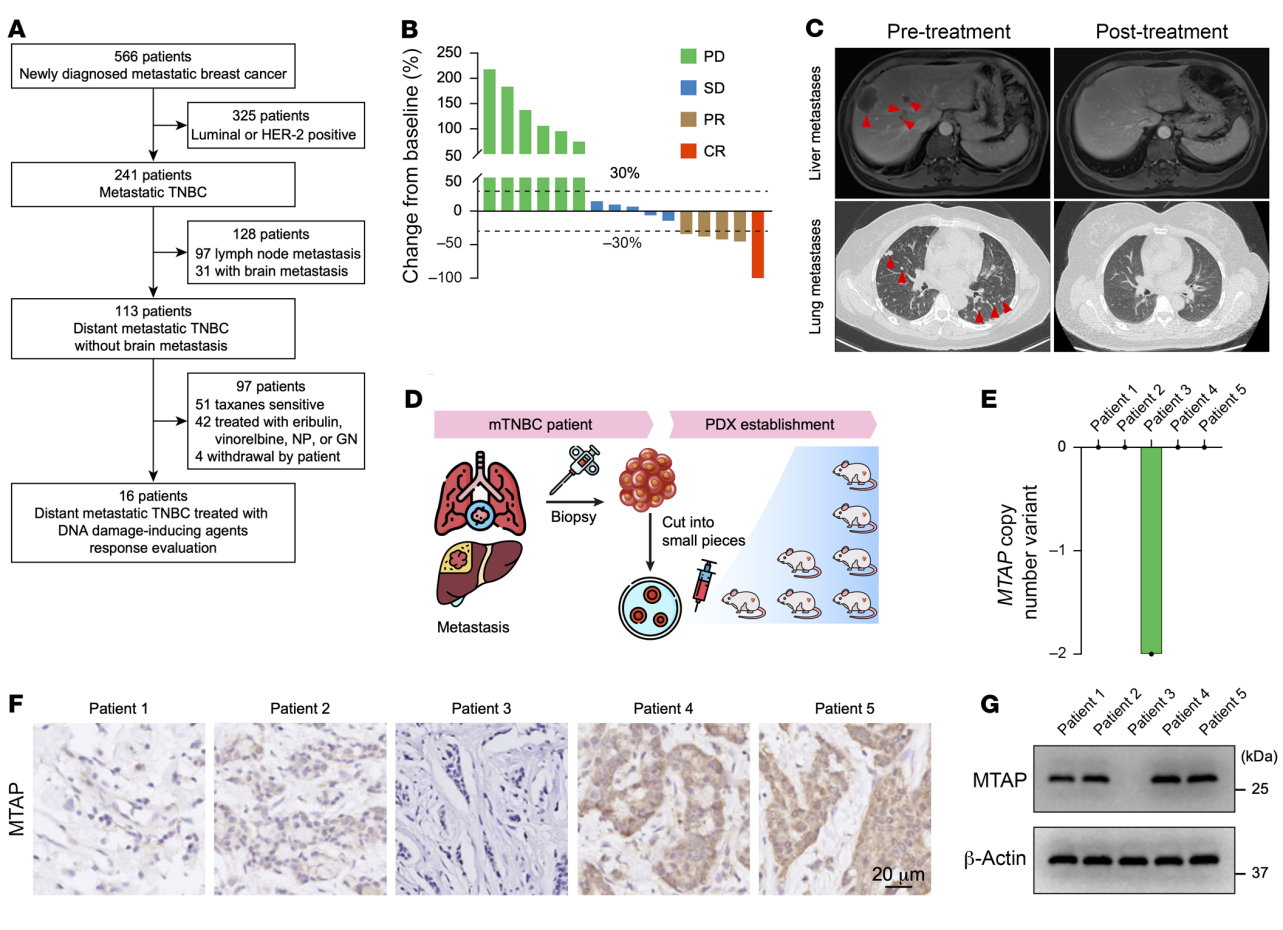
*MTAP* deficiency confers vulnerability to DNA damage–inducing agents. (**A**) Flowchart illustrating the enrollment of patients with TNBC with distant metastasis, excluding brain metastasis, who were treated with DNA damage–inducing agents. NP, vinorelbine plus platinum; GN, gemcitabine plus vinorelbine. (**B**) Percentage change from baseline of 16 distant metastatic TNBC following DNA damage–inducing agents treatment. PD, disease progression; SD, stable disease; PR, partial response; CR, complete response. (**C**) Abdominal MRI showing the liver metastases (top) and pulmonary CT scan depicting the lung metastases (bottom) of the patient that achieved CR before and after treatment. (**D**) Schematic representation of TNBC PDX model establishment. (**E**) Copy number variant of *MTAP* in the 5 TNBC analyzed by whole-genome sequencing. (**F** and **G**) Representative immunohistochemistry staining (**F**) and Western blot (**G**) of MTAP in the 5 TNBC. Scale bar: 20 μm. (**G**) The experiment was repeated 3 times, and representative blots are presented.

Patients with TNBC with distant (nonbrain) metastasis who have experienced failure of taxanes therapy are typically scheduled for either single-agent treatment (eribulin, vinorelbine, capecitabine, or gemcitabine) or combined treatment (vinorelbine plus platinum, gemcitabine plus platinum, or vinorelbine plus capecitabine), while patients with brain metastatic TNBC are usually assigned to receive surgical resection and/or radiotherapy (27, 28). To determine the potential biomarkers for response to DNA damage–inducing agents of capecitabine, gemcitabine, or gemcitabine plus platinum in patients with distant metastatic TNBC with failure of taxanes, we conducted a retrospective analysis of patients with distant metastatic TNBC treated in our center between January 1, 2021, and December 31, 2021. A total of 16 cases were ultimately enrolled for analysis (Figure 1A and Supplemental Table 1; supplemental material available online with this article; https://doi.org/10.1172/JCI188120DS1). Tumor response was assessed according to RECIST version 1.1. Among the 16 patients with distant metastatic TNBC who received DNA damage–inducing agents, 6 (37.5%) experienced disease progression, 5 (31.3%) displayed stable disease, and 4 (25%) reached partial response (Figure 1B). Strikingly, 1 of these patients achieved complete response after treatment (Figure 1C). To further investigate the underlying mechanisms of susceptibility to DNA damage–inducing agents in the complete response case, we successfully established 5 patient-derived xenografts (PDXs) from biopsy specimens obtained prior to treatment, which included 3 cases of disease progression, 1 case of stable disease, and the complete response case (Figure 1D). Genotypic assessment of these distant metastatic TNBC samples revealed WT *BRCA* status in all 5 cases, while the complete response case specifically exhibited a homozygous deletion of *MTAP* (Figure 1E), which was confirmed the lack of MTAP expression in immunohistochemistry and Western blot analysis (Figure 1, F and G). These findings suggest that *MTAP* deletion may confer increased vulnerability to genotoxic agents.

**Figure 2:**
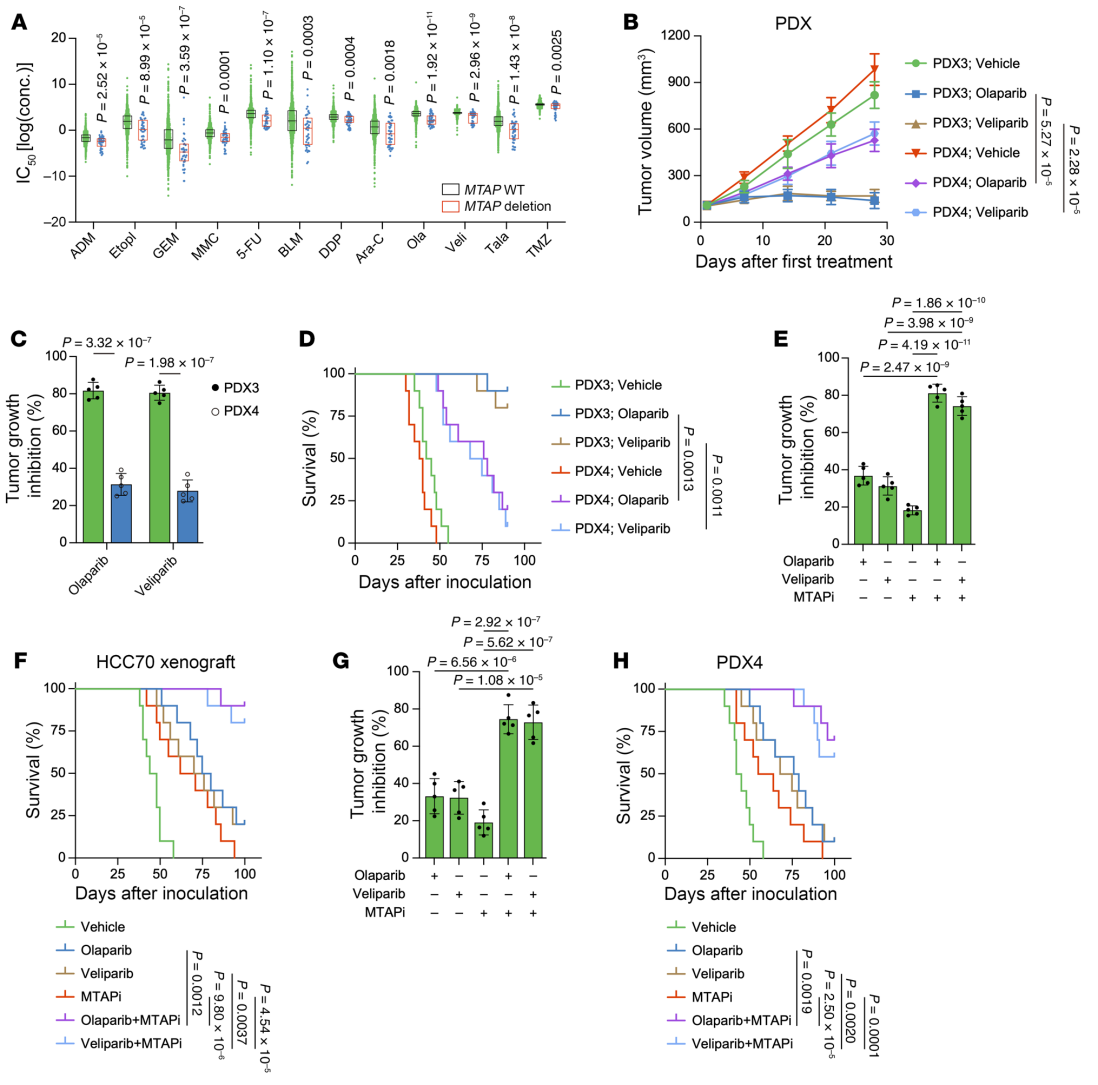
*MTAP* deficiency/inhibition renders cells susceptible to PARPi. (**A**) Predicted sensitivity to the indicated genotoxic agents for breast cancer with *MTAP* WT or deletion. (**B**–**D**) Tumor growth curves (**B**), tumor growth inhibition (**C**), and Kaplan-Meier survival curves (**D**) of PDX3 and PDX4 models treated with vehicle, olaparib (50 mg/kg), or veliparib (50 mg/kg). (**E**–**H**) Tumor growth inhibition and Kaplan-Meier survival curves of HCC70 (**E** and **F**) and PDX4 (**G** and **H**) xenograft models treated with olaparib (50 mg/kg), veliparib (50 mg/kg), or MTAPi (10 mg/kg), or the indicated combinations. (**B**, **C**, **E**, and **G**) Data are shown as the mean ± SD from 1 representative experiment of 5 mice per group. *n* = 10 mice per group in **D**, **F**, and **H**. *P* values are indicated. Significance was determined using (**A**) 2-sided Mann-Whitney *U*, (**B**) 2-way ANOVA, (**C**) unpaired *t*, (**D**, **F**, and **H**) log-rank (Mantel-Cox), or (**E** and **G**) 1-way ANOVA test.

Integrative analysis of data from The Cancer Genome Atlas (TCGA) and Genomics of Drug Sensitivity in Cancer (GDSC), utilizing the oncoPredict R package (29), revealed that patients with breast cancer with *MTAP* homologous deletion exhibited heightened sensitivity to chemotherapeutic agents, particularly PARPi (Figure 2A). To validate this observation, we knocked out *MTAP* using 1 pair of sgRNA in HCC70 and BT549 cells, both of which demonstrated elevated MTAP expression (Supplemental Figure 1, A–C). Cellular assays indicated markedly reduced cell viability in *MTAP*-null cells under genomic toxicity, supporting the hypothesis that *MTAP* deficiency confers susceptibility to genotoxic agents, with PARPi exhibiting the most pronounced effect (Supplemental Figure 1D).

Building on previous studies that demonstrated *MTAP* loss induces sensitivity to PRMT5 or MAT2A inhibition (12, 30, 31), we also investigated the effects of 2 PRMT5 inhibitors (GSK3326595 and MRTX1719) and 2 MAT2A inhibitors (AGI-24512 and AG-270) in our cellular experiments. Notably, we observed that, when combined with *MTAP* depletion, PARPi exhibited a comparable effect with PRMT5 inhibitor MRTX1719 but displayed more substantial synergistic effect than another PRMT5 inhibitor, GSK3326595, or MAT2A inhibitors (Supplemental Figure 1D). To assess the in vivo effects, we administered 4 chemotherapeutic agents orally to mice bearing subcutaneous xenografts of TNBC. Treatment, especially with PARPi, resulted in an enhanced tumor growth inhibition in *MTAP*-null xenografts (Supplemental Figure 1E). Consequently, we selected PARPi for further investigation. In our TNBC PDX models, we also observed higher sensitivity to PARPi in PDX3, which harbors *MTAP* deletion, compared with PDX4, which expresses elevated levels of MTAP (Figure 2, B–D).

Given that only approximately 4% of patients with breast cancer harbor *MTAP* homozygous deletion (http://www.cbioportal.org), we investigated the potential synergy between MTAP inhibition and PARPi in TNBC. Indeed, pharmacologic MTAP inhibition with a MTAP inhibitor (MTAPi), methylthio-DADMe-Immucillin-A, markedly sensitized MTAP-expressing TNBC cells to PARPi (Supplemental Figure 1, F–K). Furthermore, the combination efficacy of MTAPi and PARPi was reproduced in mouse xenograft models (Figure 2, E–H, and Supplemental Figure 1, L and M). The combination drug treatment was well tolerated, as evidenced by the absence of any notable effects on mouse body weight as well as a lack of substantial systemic toxicity observed in TUNEL-stained sections of the heart, liver, and kidneys (Supplemental Figure 2, A–F). To further assess the toxicity profile of the MTAPi and PARPi combination in normal cells, we obtained 2 human mammary epithelial cell lines expressing MTAP and conducted MTS assays. Consistent with the findings from the mouse models, MTS data indicated that treatment with MTAPi did not render normal cells susceptible to PARPi (Supplemental Figure 2, G–I).

### PARPi suppresses MAT2A expression by inducing METTL16 autoinhibition-mediated intron retention.

Recent studies have demonstrated that the inhibition of MAT2A results in a selective antiproliferative effect in cancers characterized by *MTAP* deletion (13–15, 30). It is well established that N^6^-adenosine methyltransferase METTL16 promotes MAT2A expression by enhancing splicing of its mRNA retained intron, particularly under SAM depletion conditions (32–34). Our preliminary investigation indicated that activation of ATM following DNA damage phosphorylates METTL16, leading to a conformational change that decreases its affinity for substrate RNA (35). Therefore, we hypothesize that PARPi may function like MAT2A inhibitors by suppressing MAT2A’s expression, thereby exhibiting synergistic effects with *MTAP* deletion/inhibition.

**Figure 3:**
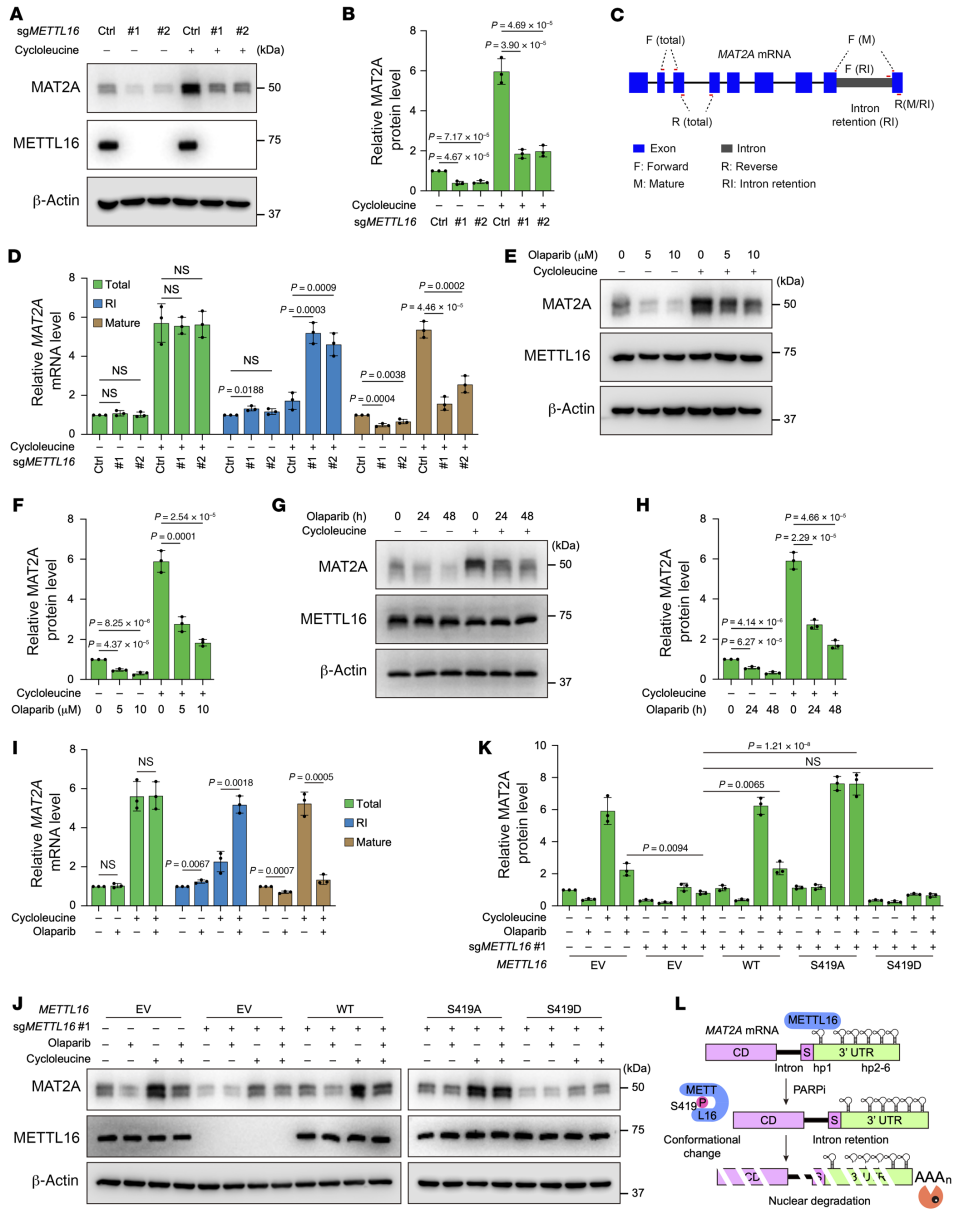
PARPi regulates *MAT2A* intron retention by mediating METTL16 phosphorylation within Ser419. (**A** and **B**) Representative Western blots (**A**) and quantitation (**B**) showing levels of MAT2A and METTL16 in HCC70 cells expressing control or *METTL16* sgRNAs with or without cycloleucine (20 mM) treatment. (**C**) Schematic representation of RT-qPCR assay primers designed for total, intron retention (RI), or mature *MAT2A* mRNA. (**D**) RT-qPCR analysis of total, intron retention, and mature *MAT2A* mRNA levels in HCC70 cells with or without *METTL16* KO treated with vehicle or cycloleucine (20 mM). (**E**–**H**) Representative Western blots and quantitation showing levels of MAT2A and METTL16 in HCC70 cells treated with the indicated dose of olaparib for 24 hours (**E** and **F**) or the indicated time period of olaparib at 5 μM (**G** and **H**). (**I**) RT-qPCR analysis of total, intron retention, and mature *MAT2A* mRNA levels in HCC70 cells treated with olaparib (5 μM), or cycloleucine (20 mM), or their combination. (**J** and **K**) Representative Western blots (**J**) and quantitation (**K**) showing levels of MAT2A and METTL16 in HCC70 cells expressing the indicated vectors treated with olaparib (5 μM), cycloleucine (20 mM), or their combination. (**L**) Schematic representation of PARPi downregulation of MAT2A expression by attenuation of its mRNA splicing through METTL16 conformational change induced by phosphorylation within Ser419. (**A**, **E**, **G**, and **J**) The experiment was repeated 3 times, and representative blots are presented. (**B**, **D**, **F**, **H**, **I**, and **K**) Data are shown as the mean ± SD from 3 independent experiments. *P* values are indicated. Significance was determined using (**B**, **D**, **F**, **H**, **I**, and **K**) 1-way ANOVA test.

To elucidate the role of METTL16 in regulating MAT2A expression, we employed 2 independent sgRNAs to KO *METTL16*. The results indicated that depletion of *METTL16* led to a substantial reduction in MAT2A protein levels, while MAT2B and MAT1A levels remained unaffected, particularly upon SAM depletion conditions, which induced MAT2A expression and were imitated by MAT2A inhibitor cycloleucine treatment (Figure 3, A and B, and Supplemental Figure 3, A and B). Furthermore, reintroduction of WT *METTL16* into *METTL16*-null cells restored MAT2A protein levels, whereas neither the RNA binding motif deletion mutant (MR2) nor the catalytically inactive mutant (F187G) was able to achieve this effect (Supplemental Figure 3, C–F). Expectedly, we found an increase in premature *MAT2A* mRNA with retained introns and a concomitant reduction in mature *MAT2A* mRNA, while total mRNA levels remained unchanged in *METTL16*-KO cells (Figure 3, C and D). Furthermore, we observed a strong positive correlation for the protein expressions of MAT2A and METTL16 in both cell lines and tumors (Supplemental Figure 3, G and H).

Upon treatment with DNA damage–inducing agents, we found that PARPi markedly induced METTL16 phosphorylation while concurrently reducing MAT2A expression (Supplemental Figure 4, A–C), results that align with the functional results presented in Supplemental Figure 1, D and E. Additionally, we noticed that PARPi treatment led to a dose- and time-dependent reduction in MAT2A protein levels and disrupted *MAT2A* mRNA splicing of retaining introns (Figure 3, E–I, and Supplemental Figure 4, D–H). By reintroducing WT *METTL16*, along with a phosphorylation-defective mutant (S419A) and a phosphorylation-mimicking mutant (S419D), into *METTL16*-null cells, we found that WT *METTL16*, but not the S419D mutant, could restore MAT2A levels under PARPi treatment. Notably, reconstitution with the S419A mutant resulted in a further increase in MAT2A levels following PARPi treatment (Figure 3, J and K, and Supplemental Figure 4, I and J). We also observed consistent findings of PARPi regulating MAT2A levels in another cell line (Supplemental Figure 5).

Together, these results suggest that PARPi inhibits MAT2A by attenuating its mRNA splicing, which was attributed to PARPi-mediated conformational change and autoinhibition of METTL16 (Figure 3L).

### MTAPi synergizes with PARPi by diminishing SAM.

**Figure 4:**
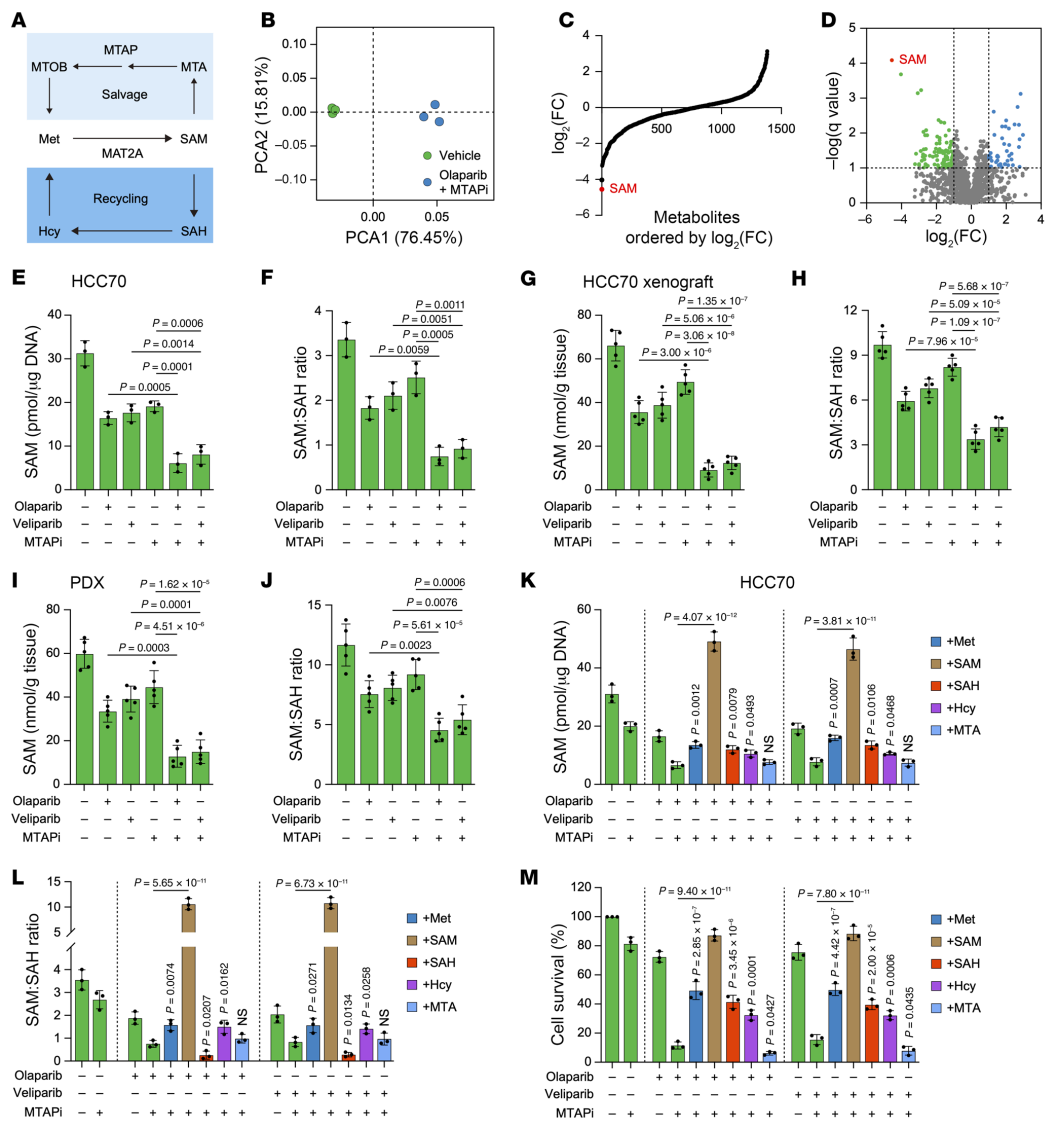
PARPi and MTAPi in combination effectively kill tumor cells by reducing SAM. (**A**) Schematic representation of the methionine recycling and salvage pathways. (**B**) Principal component analysis (PCA) score scatter plots of vehicle and olaparib combined with MTAPi groups. (**C** and **D**) Waterfall plot (**C**) and volcano plot (**D**) of intracellular metabolite level analysis using untargeted LC-MS in HCC70 cells treated with vehicle or olaparib (2 μM) combined with MTAPi (1 μM). (**E** and **F**) Intracellular SAM (**E**) and SAM/SAH ratio (**F**) of HCC70 cells treated with olaparib (2 μM), veliparib (2 μM), or MTAPi (1 μM) or the indicated combinations. (**G**–**J**) Intratumor SAM and SAM/SAH ratio of HCC70 (**G** and **H**) and PDX4 (**I** and **J**) xenograft tumors treated with olaparib (50 mg/kg), veliparib (50 mg/kg), or MTAPi (10 mg/kg) or the indicated combinations. Data are shown as the mean ± SD from 1 representative experiment of 5 mice per group. (**K**–**M**) Intracellular SAM (**K**), SAM/SAH ratio (**L**), and colony formation assay (**M**) of HCC70 cells treated with olaparib, veliparib, or MTAPi or the indicated combinations and supplemented with 100 μM Met, SAM, SAH, Hcy, or MTA. (**E**, **F**, and **K**–**M**) Data are shown as the mean ± SD from 3 independent experiments. *P* values are indicated. Significance was determined using (**E**–**M**) 1-way ANOVA test.

MTAP is a crucial enzyme in the Met salvage pathway that plays a pivotal role in recycling the 1-carbon unit lost back into the Met cycle to regenerate SAM (Figure 4A) (10, 11). MAT2A synthesizes SAM from ATP and Met. Our aforementioned data suggested that PARPi suppressed MAT2A by inhibiting its mRNA splicing. In light of this, we asked whether MTAPi combined with PARPi effectively kills tumor cells by disrupting the Met pathway.

We conducted a broad, untargeted liquid chromatograph–mass spectrometer–based (LC-MS–based) metabolomic assessment of intracellular metabolite levels in HCC70 cells without treatment or treated with olaparib and MTAPi. The analysis revealed that, among 1,384 annotated metabolites detected, SAM exhibited the most substantial decrease in abundance in cells treated with the olaparib and MTAPi combination compared with the vehicle control (Figure 4, B–D, and Supplemental Table 2). Indeed, the intracellular or intratumor SAM and SAM/SAH ratio were markedly reduced in both cell and mouse models following MTAPi and PARPi treatment (Figure 4, E–J, and Supplemental Figure 6, A–D).

**Figure 5:**
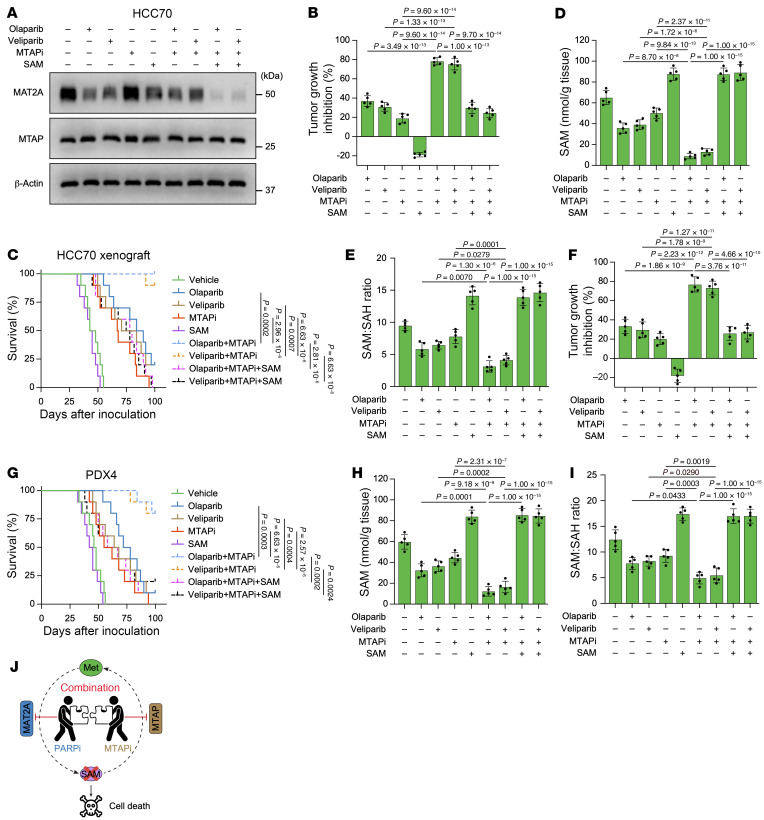
PARPi combined with MTAPi markedly inhibit tumor growth through diminishing SAM. (**A**) Representative Western blots showing levels of MAT2A and MTAP in HCC70 cells treated with olaparib (2 μM), veliparib (2 μM), MTAPi (1 μM), or SAM (100 μM), or the indicated combinations. The experiment was repeated 3 times, and representative blots are presented. (**B**–**I**) Tumor growth inhibition, Kaplan-Meier survival curves, intratumor SAM, and SAM/SAH ratio of HCC70 (**B**–**E**) and PDX4 (**F**–**I**) xenograft tumors treated with olaparib, veliparib, or MTAPi or the indicated combinations. Olaparib and veliparib were used at 50 mg/kg and MTAPi was used at 10 mg/kg, intraperitoneally, 5 times per week. SAM was administered at 10 mg/kg subcutaneously each day. (**B**, **D**, **E**, **F**, **H**, and **I**) Data are shown as the mean ± SD from 1 representative experiment of 5 mice per group. *n* = 10 mice per group in **C** and **G**. *P* values are indicated. Significance was determined using (**B**, **D**, **E**, **F**, **H**, and **I**) 1-way ANOVA test or (**C** and **G**) log-rank (Mantel-Cox) test. (**J**) Schematic representation of PARPi exhibiting synergistic effect with MTAP inhibition.

To investigate the role of SAM in cell or tumor inhibition by MTAPi and PARPi, we performed combination treatment alongside rescue experiments utilizing metabolites of Met itself, SAM, S-adenosylhomocysteine (SAH), homocysteine (Hcy), or 5-MTA. In both HCC70 and BT549 cells, the SAM depletion, SAM/SAH ratio, and cell death induced by the combination of MTAPi and PARPi was effectively mitigated by supplementation with Met, SAM, SAH, or Hcy, with SAM supplementation demonstrating the most pronounced effect (Figure 4, K–M, and Supplemental Figure 6, E–G). Conversely, MTA supplementation did not markedly alter SAM or the SAM/SAH ratio but resulted in reduced cell survival (Figure 4, K–M, and Supplemental Figure 6, E–G), corroborating prior findings (36, 37). Meanwhile, expression of MAT2A was further decreased but not induced following SAM supplementation (Figure 5A and Supplemental Figure 7A). Notably, the administration of SAM in mice reversed the tumor growth inhibition and survival benefits associated with the depleted SAM and SAM/SAH ratio contributed by combined treatment with MTAPi and PARPi (Figure 5, B–I, and Supplemental Figure 7, B–E).

Thus, the in vitro and in vivo results indicate that the reduction in SAM is an on-target effect resulting from the strong synergy between MTAPi and PARPi (Figure 5J).

### Reduced SAM prompts PARPi-induced DNA damage by blunting MRE11 recruitment and end resection.

SAM serves as the primary methyl donor in biosynthetic methylation reactions, and accumulating evidence highlights the pivotal role of protein methylation in DNA repair (38–40). Accordingly, we sought to investigate whether the substantial reduction of SAM induced by the combination treatment would impair methylation within cells, ultimately hindering DNA damage repair lead by PARPi.

**Figure 6:**
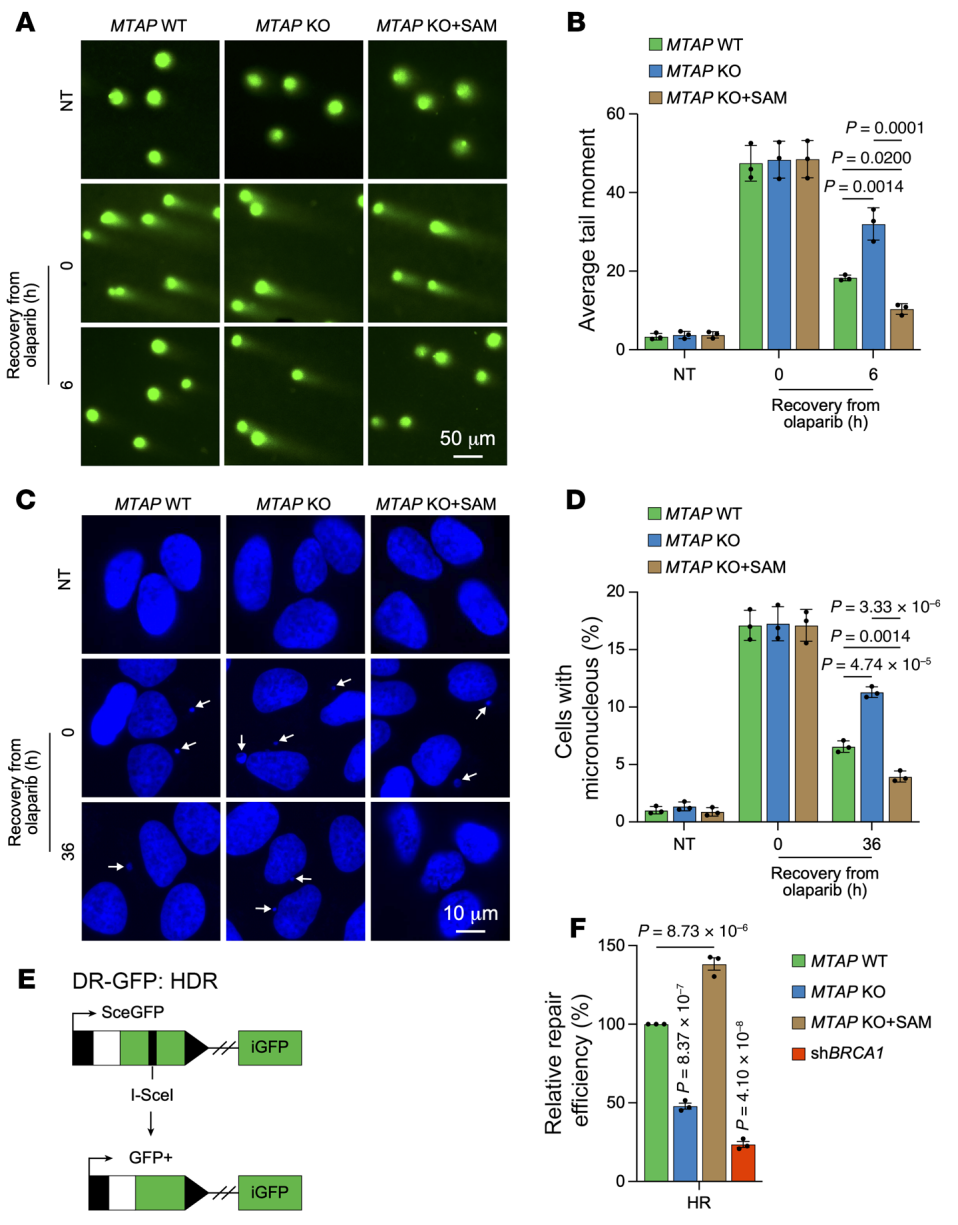
SAM depletion caused by MTAP and PARP inhibition attenuates DNA repair. (**A**–**D**) Representative micrographs and quantitation of neutral comet assay (**A** and **B**) and micronucleus assay (**C** and **D**) in the indicated cells without treatment or recovery at 0 hours, 6 hours, or 36 hours after olaparib (20 μM) treatment. (**A** and **C**) The experiment was repeated 3 times, and representative micrographs are presented. Scale bar: 50 μm (**A**); 10 μm (**C**). (**E**) Schematic representation of the DR-GFP reporter system. (**F**) Relative HR repair efficiency in the indicated cells. sh*BRCA1* was used as a positive control. (**B**, **D**, and **F**) Data are shown as the mean ± SD from 3 independent experiments. *P* values are indicated. Significance was determined using (**B**, **D**, and **F**) 1-way ANOVA test.

First, we measured the effect of *MTAP* depletion combined with PARPi on DNA damage using the neutral comet assay and micronucleus assay. Our data showed that cells with *MTAP* deficiency displayed longer tail moments and an increased micronucleus percentage compared with control cells at 6 hours or 36 hours of recovery after olaparib treatment, although no marked differences were observed at 0 hours of recovery after olaparib treatment (Figure 6, A–D), suggesting a compromised in DNA repair mechanisms. This difference could be rescued by SAM supplementation (Figure 6, A–D), confirming that the effect of diminishing SAM is on target. These results suggest that SAM depletion caused by *MTAP* loss combined with PARPi has a detrimental impact on DNA repair.

**Figure 7:**
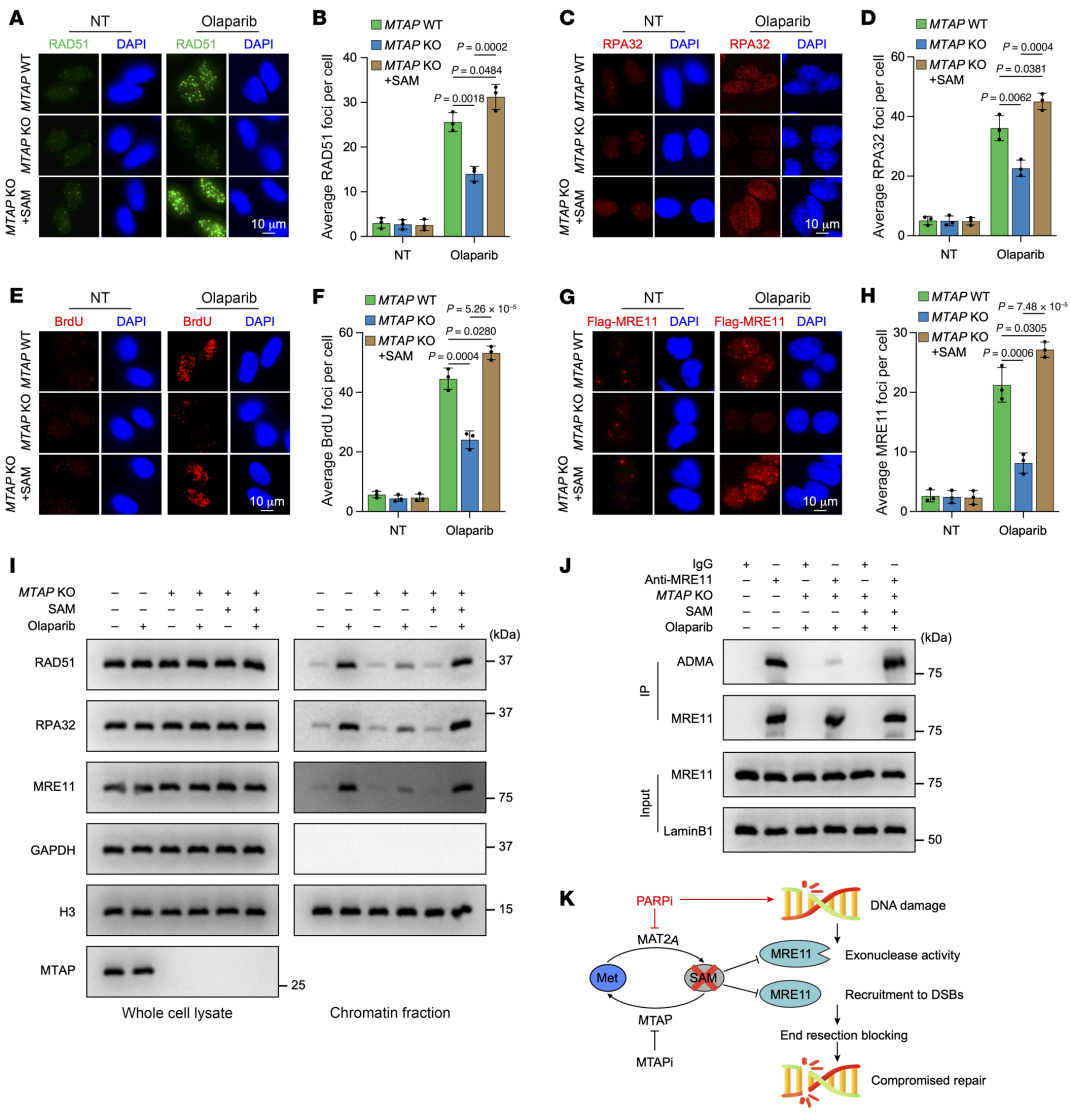
SAM depletion attenuates DNA repair by impairing methylation of MRE11-mediated end resection and recruitment. (**A**–**H**) Representative micrographs and quantitation for RAD51 (**A** and **B**), RPA32 (**C** and **D**), BrdU (**E** and **F**), or MRE11 (**G** and **H**) foci formation in the indicated cells without treatment or recovery at 1 hour after olaparib (20 μM) treatment. (**I**) Representative Western blots immunoblotted with the indicated antibodies in whole-cell lysate and chromatin fraction of the indicated cells without treatment or recovery at 1 hour after olaparib (20 μM) treatment. (**J**) Representative Western blots showing methylation levels of MRE11 in cells with the indicated treatment. ADMA, asymmetric dimethyl-arginine antibody. (**K**) Schematic representation of a feed-forward loop between low SAM levels resulting from treatment with PARPi, MTAPi combined with MR, and PARPi-induced DNA damage. (**A**, **C**, **E**, **G**, **I**, and **J**) The experiment was repeated 3 times, and representative micrographs/blots are presented. (**B**, **D**, **F**, and **H**) Data are shown as the mean ± SD from 3 independent experiments. *P* values and scale bars are indicated. Significance was determined using (**B**, **D**, **F**, and **H**) 1-way ANOVA test.

We utilized a DR-GFP reporter system (Figure 6E) to further evaluate the impact of SAM depletion on DNA DSBs. The results demonstrated that *MTAP* KO sharply decreased HR repair efficiency, which can also be reversed by SAM supplementation (Figure 6F). We next studied the underlying mechanism. First, we examined the recruitment of PALB2, CtIP, BRCA1, and NBS1 to DNA damage sites after olaparib treatment. The results indicated no marked changes in PALB2, CtIP, BRCA1, and NBS1 foci formation in *MTAP*-depleted cells when exposed to olaparib (Supplemental Figure 8, A–H). However, there was a notable decrease of RAD51 and RPA32 foci formation in *MTAP*-depleted cells following olaparib treatment (Figure 7, A–D). This decrease of RAD51 and RPA32 foci formation could be abrogated by SAM addition. Decreased RPA32 foci formation implies lowered single-stranded DNA (ssDNA) formation by end resection. We validated this using a 5-bromo-2′-deoxyuridine (BrdU) incorporation assay, a widely used method for detecting ssDNA produced during DNA end resection (41). After 1 hour of olaparib treatment, BrdU signals were sharply decreased in *MTAP*-KO cells but could be rescued with SAM supplementation (Figure 7, E and F), further supporting an inhibitory role for low levels of SAM induced by *MTAP* deficiency in end resection.

Since no marked changes in BRCA1 or CtIP recruitment were observed, and given the critical role of MRE11 in initiating end resection to generate short stretches of ssDNA, we focused on examining MRE11 foci formation. The results showed that the foci formation of MRE11 was markedly reduced in *MTAP*-null cells with olaparib treatment, and that this reduction could be recovered by SAM supplementation (Figure 7, G and H). Notably, the combined treatment did not alter the expression levels among RAD51, RPA32, MRE11, PALB2, CtIP, BRCA1, or NBS1, nor did it affect the interaction within the MRE11-RAD50-NBS1 complex (Supplemental Figure 8, I and J). In support of the immunofluorescence results, analysis of chromatin-enriched fractions revealed that *MTAP* KO decreases the accumulation of RAD51, RPA32, and MRE11, which could be rescued by SAM supplementation, but not PALB2, CtIP, BRCA1, or NBS1, in chromatin-enriched protein extracts after olaparib treatment (Figure 7I and Supplemental Figure 8K). Collectively, these results suggest that *MTAP* deletion combined with PARPi leads to a reduction in SAM levels, thereby impairing DNA repair by hindering the recruitment of MRE11 to DSBs and attenuating end resection.

MRE11 contains a glycine- and arginine-rich sequence termed the GAR motif (42). Methylation of this motif in MRE11 enables its exonuclease activity during end resection and its ability to signal DNA damage (43, 44). Therefore, we hypothesized that depleted SAM induced by *MTAP* KO combined with PARPi represses recruitment of MRE11 to DSBs and end resection through attenuating the methylation of MRE11. Indeed, analysis using an asymmetric dimethyl-arginine antibody revealed that the methylation levels of MRE11 were markedly reduced in *MTAP*-null cells treated with olaparib (Figure 7J), thereby confirming our hypothesis.

Therefore, these data strongly indicate a feed-forward loop between depleted SAM and compromised DNA repair caused by *MTAP* deletion combined with PARPi and PARPi-induced DNA damage (Figure 7K).

### MR reinforces the antitumor efficacy of PARPi and MTAP deficiency/inhibition by depleting SAM.

The essential amino acid Met, which must be obtained through exogenous sources, plays a crucial role in various anabolic processes that promote cancer cell growth and proliferation (45, 46). Restriction of dietary Met can further deplete intracellular SAM levels, potentially augmenting the combinatorial effects of *MTAP* deficiency/inhibition and PARPi. To address this, we conducted cellular and mouse experiments under Met deprivation conditions.

**Figure 8:**
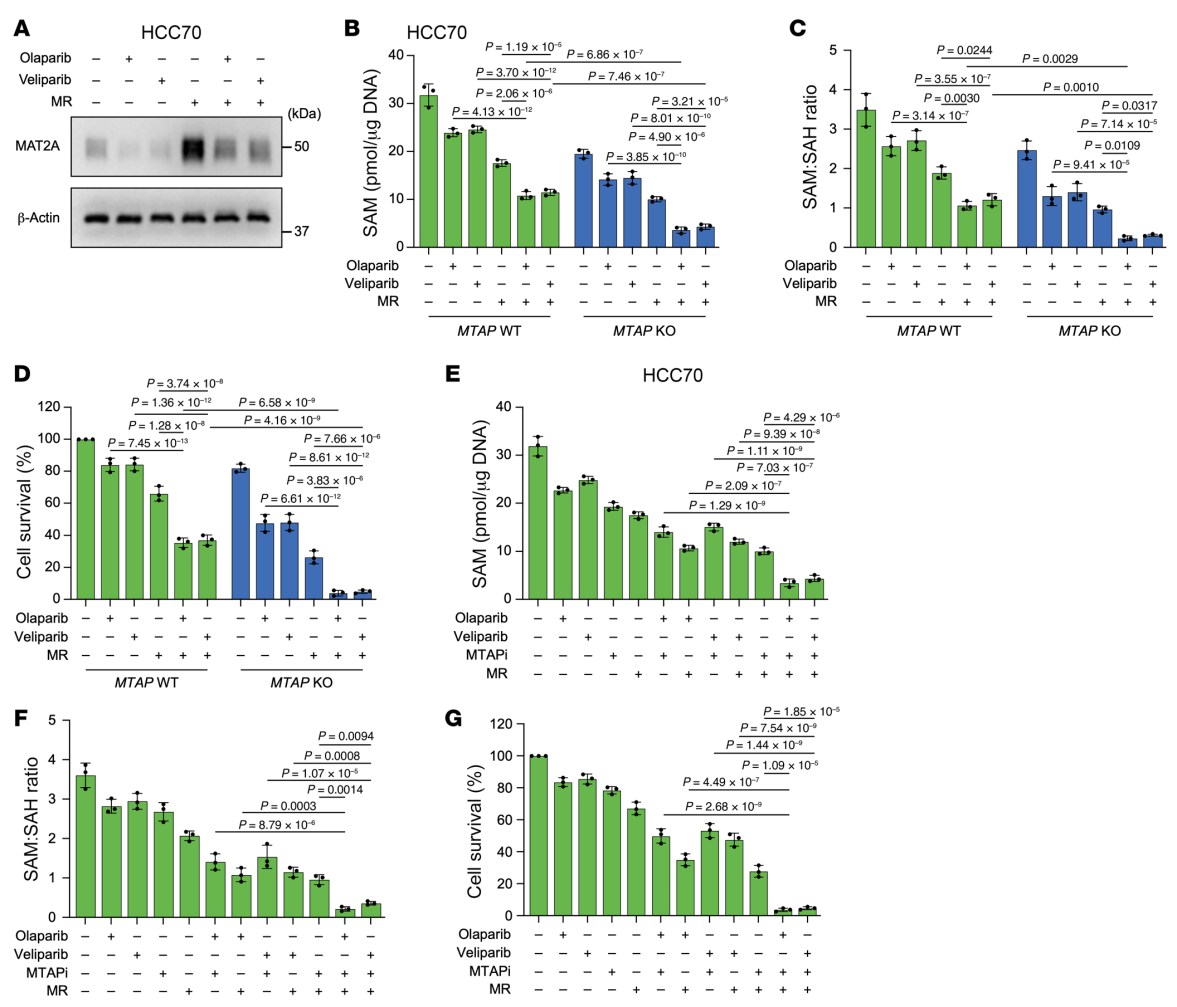
Methionine restriction augments the combination efficacy of *MTAP* deficiency/inhibition and PARPi. (**A**) Representative Western blots showing levels of MAT2A in HCC70 cells treated with olaparib (2 μM), veliparib (2 μM), or methionine restriction (MR) (20%) or the indicated combinations. The experiment was repeated 3 times, and representative blots are presented. (**B**–**D**) Intracellular SAM (**B**), SAM/SAH ratio (**C**), and colony formation assay (**D**) of HCC70 cells with or without *MTAP* deletion treated with olaparib, veliparib, or MR or the indicated combinations. (**E**–**G**) Intracellular SAM (**E**), SAM/SAH ratio (**F**), and colony formation assay (**G**) of HCC70 cells treated with olaparib, veliparib, MTAPi, or MR or the indicated combinations. (**B**–**G**) Data are shown as the mean ± SD from 3 independent experiments. *P* values are indicated. Significance was determined using (**B**–**G**) 1-way ANOVA test.

**Figure 9:**
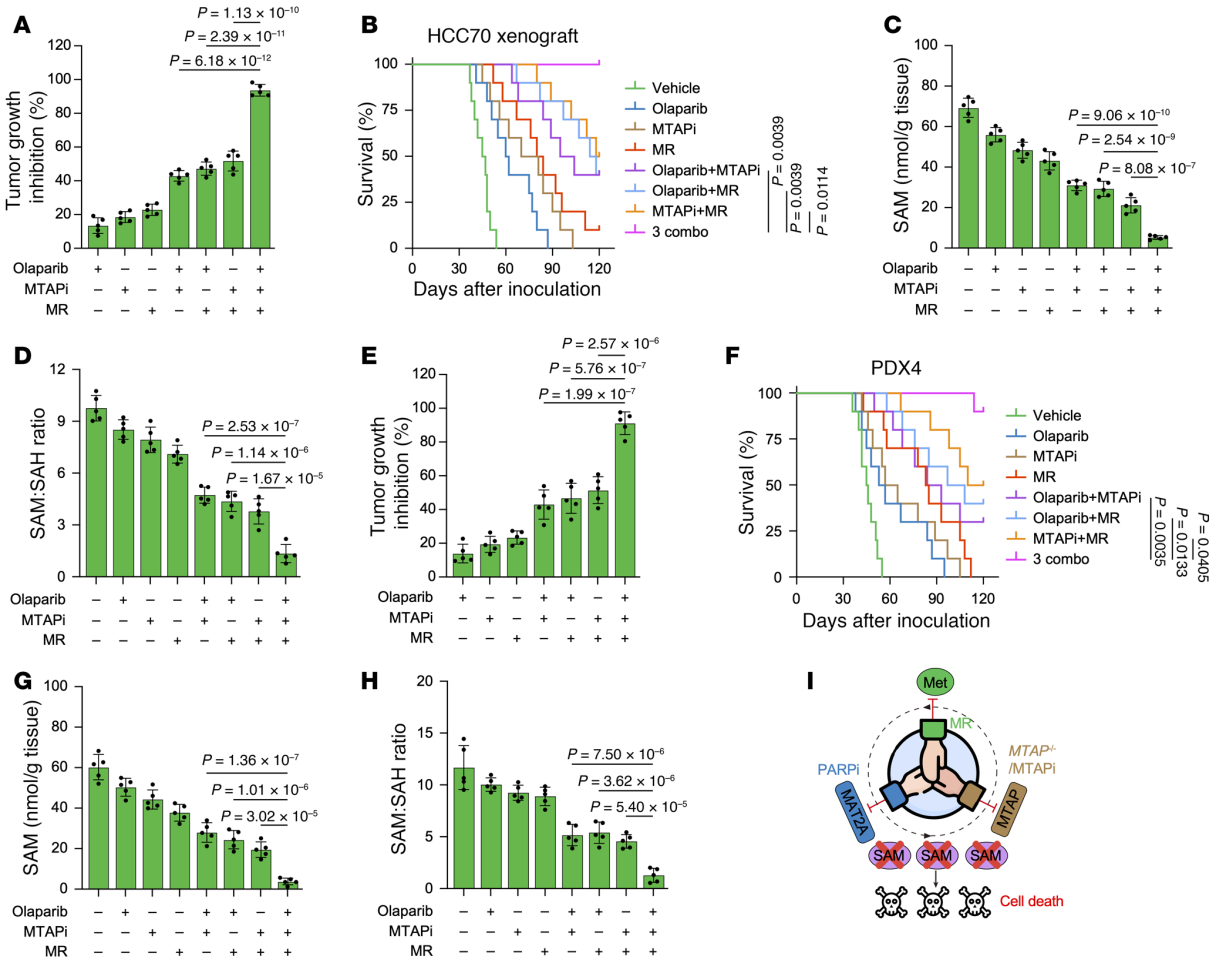
MR boosts the antitumor effect of MTAP and PARP inhibition in vivo. (**A**–**H**) Tumor growth inhibition, Kaplan-Meier survival curves, intratumor SAM, and SAM/SAH ratio of HCC70 (**A**–**D**) and PDX4 (**E**–**H**) xenograft tumors treated with olaparib, MTAPi, MR, or the indicated combinations. All drugs were used at 10 mg/kg, intraperitoneally, 5 times per week. The control diet contained 0.86% methionine, and methionine restriction diet contained 0.12% methionine. (**A**, **C–E**, **G**, and **H**) Data are shown as the mean ± SD from 1 representative experiment of 5 mice per group. *n* = 10 mice per group in **B** and **F**. *P* values are indicated. Significance was determined using (**A**, **C**–**E**, **G**, and **H**) 1-way ANOVA or (**B** and **F**) log-rank (Mantel-Cox) test. (**I**) Schematic representation of MR enhancing the combination effect of PARPi and *MTAP* deficiency/inhibition through depletion of SAM.

We observed that PARPi effectively blocked the upregulation of MAT2A induced by MR (Figure 8A and Supplemental Figure 9A). In vitro experiments demonstrated that MR enhances the efficacy of the combination treatment of PARPi and *MTAP* deletion or inhibition in both HCC70 and BT549 cells by depleting SAM and the SAM/SAH ratio (Figure 8, B–G, and Supplemental Figure 9, B–G). Moreover, the combinatorial effect of PARPi and MTAPi was further potentiated by MR in mouse models (Figure 9, A–H, and Supplemental Figure 10, A–D). Notably, the triple combination–treated group did not show any body weight loss or reduction in food intake, and more importantly, at the end of the study, the TUNEL staining examination of tissues did not identify any treatment-related impairment (Supplemental Figure 10, E–M). These results further support our hypothesis that MR heightens the combination efficacy of PARPi and *MTAP* deficiency/inhibition by further lowering SAM levels (Figure 9I).

### Lower dose of PARPi combined with MTAP deficiency/inhibition remarkably killed tumor cells in brain metastatic TNBC.

BrM is a serious, life-threatening event for patients with TNBC (1, 3, 4). Multiple studies indicate that BrM possesses unique metabolic characteristics compared with metastasis in other sites, as they experience severe depletion of amino acids relative to the plasma in both the brain interstitial fluid and cerebrospinal fluid (47–49). On the other way, the amino acid–depleted environment of the interstitial fluid or cerebrospinal fluid might create targetable metabolic dependencies in BrM.

Our results suggest that MR could enhance the combination efficacy of PARPi and *MTAP* deficiency/inhibition in vitro and in vivo. Strikingly, the brain is an inherently Met-limited environment, which can synergize with PARPi and *MTAP* deficiency/inhibition, thereby improving treatment outcomes for brain metastatic TNBC. Given that veliparib is well documented as crossing the intact blood-brain barrier (50, 51), we employed veliparib for further investigation. Additionally, in vivo pharmacokinetic studies of MTAPi have demonstrated their effective penetration of the blood-brain barrier (Supplemental Figure 11A and Supplemental Table 3).

**Figure 10:**
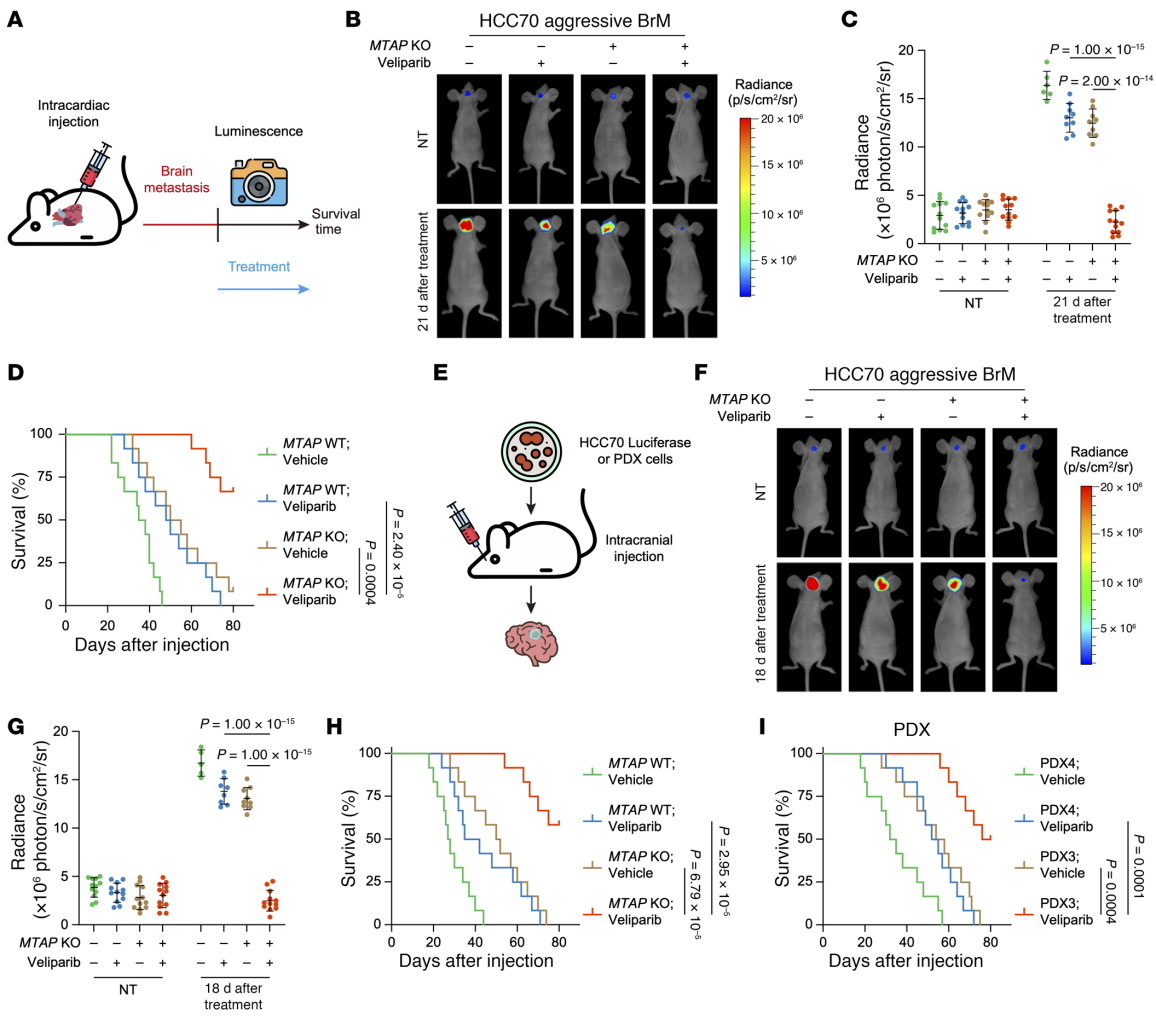
*MTAP* deficiency confers a therapeutic vulnerability to lower dose of PARPi in brain metastatic TNBC. (**A**) Schematic representation of treatment scheme for brain metastatic TNBC. (**B**–**D**) Representative in vivo bioluminescence imaging (**B**), quantification of radiance (**C**) prior to treatment or 21 days after treatment, and Kaplan-Meier survival curves (**D**) of intracardiac injection mouse models of *MTAP* WT or deleted HCC70 aggressive BrM cells, treated with vehicle or veliparib (10 mg/kg). (**E**) Schematic representation of intracranial injection of HCC70 luciferase or PDX cells. (**F**–**H**) Representative in vivo bioluminescence imaging (**F**), quantification of radiance (**G**) prior to treatment or 18 days after treatment, and Kaplan-Meier survival curves (**H**) of intracranial injection mouse models of *MTAP* WT or deleted HCC70 aggressive BrM cells, treated with vehicle or veliparib (10 mg/kg). (**I**) Kaplan-Meier survival curves of PDX3 and PDX4 intracranial injection mouse models treated with vehicle or veliparib (10 mg/kg). (**C** and **G**) Data are shown as the mean ± SD from 1 representative experiment. *n* = 12 mice per group in **D**, **H**, and **I**. *P* values are indicated. Significance was determined using (**C** and **G**) 1-way ANOVA or (**D**, **H**, and **I**) log-rank (Mantel-Cox) test.

We established TNBC BrM cell lines by in vivo selection using the HCC70 cells expressing luciferase, with or without *MTAP* deletion (Supplemental Figure 11B). The aggressive HCC70 BrM cells generated multifocal lesions in the cerebrum, cerebellum, and brain stem of mice, whereas the indolent BrM cells failed to form detectable brain metastases over a period of approximately 12 weeks (Supplemental Figure 11, C–H). Three weeks after intracardiac injection of aggressive HCC70 BrM cells, we administered a lower dose of veliparib to mice bearing aggressive BrM with or without *MTAP* KO and monitored the signal of BrM cells by bioluminescence imaging (BLI) (Figure 10A). The results showed that the lower dose of veliparib monotherapy dramatically relieved the burden of BrM and improved survival in mice with *MTAP*-deficient cells (Figure 10, B–D). Furthermore, we conducted orthotopic tumor metastasis assays using intracranial injection (Figure 10E). Consistently, mice bearing aggressive BrM or PDX cells (PDX3) with depleted *MTAP* markedly benefited from the lower-dose veliparib treatment, with reduced BrM burden and prolonged survival (Figure 10, F–I).

**Figure 11:**
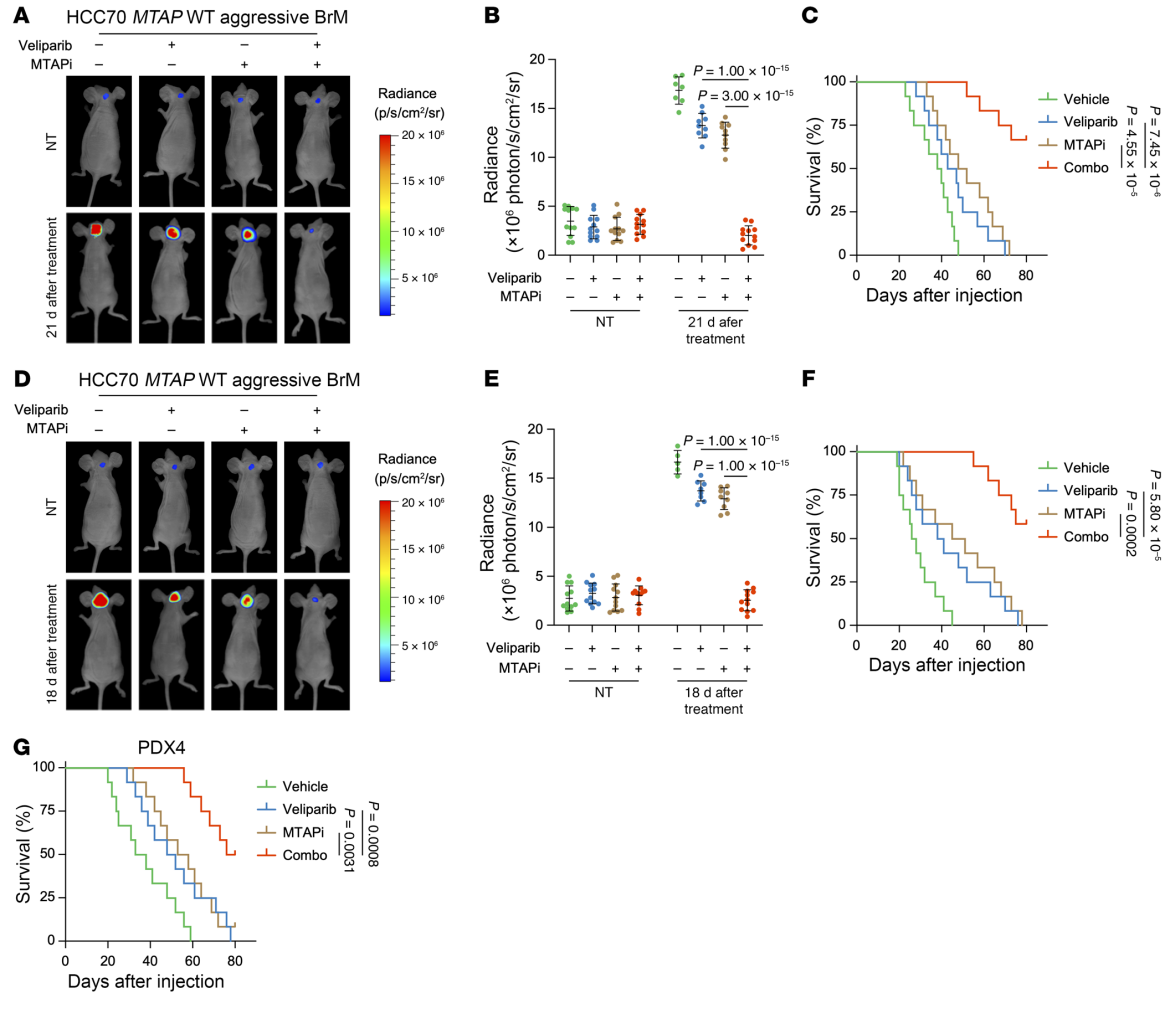
MTAPi combined with low-dose PARPi profoundly benefits brain metastatic TNBC. (**A**–**C**) Representative in vivo bioluminescence imaging (**A**), quantification of radiance (**B**) prior to treatment or 21 days after treatment, and Kaplan-Meier survival curves (**C**) of intracardiac injection mouse models of *MTAP* WT HCC70 aggressive BrM cells, treated with vehicle, veliparib (10 mg/kg), or MTAPi (10 mg/kg) or their combination. (**D**–**F**) Representative in vivo bioluminescence imaging (**D**), quantification of radiance (**E**) prior to treatment or 18 days after treatment, and Kaplan-Meier survival curves (**F**) of intracranial injection mouse models of *MTAP* WT HCC70 aggressive BrM cells, treated with vehicle, veliparib (10 mg/kg), or MTAPi (10 mg/kg) or their combination. (**G**) Kaplan-Meier survival curves of PDX4 intracranial injection mouse models treated with vehicle, veliparib (10 mg/kg), or MTAPi (10 mg/kg) or their combination. (**B** and **E**) Data are shown as the mean ± SD from 1 representative experiment. *n* = 12 mice per group in **C**, **F**, and **G**. *P* values are indicated. Significance was determined using (**B** and **E**) 1-way ANOVA or (**C**, **F**, and **G**) log-rank (Mantel-Cox) test.

For TNBC cells expressing MTAP, we administrated a combination therapy of lower-dose veliparib and MTAPi. In line with the findings in *MTAP*-deficient cells, the combination of lower-dose veliparib and MTAPi markedly reduced the burden of BrM and enhanced survival in mice with MTAP-expressing cells, as demonstrated in both intracardiac injection and intracranial injection models (Figure 11).

Taken together, these data demonstrate that *MTAP* deficiency/inhibition combined with lower-dose PARPi provides an effective treatment strategy for brain metastatic TNBC.

## Discussion

TNBC disproportionately affects younger women and is marked by heightened susceptibility to relapse, more frequent metastasis, and inferior survival compared with other breast cancer subtypes (1, 4). The intricate molecular heterogeneity of TNBC, along with the lack of easily identifiable targets, poses critical challenges in the pursuit of effective targeted therapies (2–4). Encouraging clinical trials involving PARPi in *BRCA*-deficient metastatic breast cancer have demonstrated the viability of leveraging DNA repair deficiencies for targeted treatment within this subset of breast cancer (17). Indeed, extensive endeavors are currently in progress to ascertain the complete scope of the BRCAness phenotype in TNBC. Accordingly, the identification of novel targets and modulators of HR repair to augment the effectiveness and expand the applicability of PARPi stands as a paramount objective in the field of therapeutics. However, the crosstalk between metabolism and PARPi-induced DNA damage repair is under explored.

Metabolic phenotypes arise from the intricate interplay between genes and the environment. Determinants of these phenotypes include the genomic encoding of metabolic genes along with their sequence variants, the regulation of metabolic enzyme activity at both transcriptional and allosteric levels, and the availability of nutrients (45, 46). Regardless of the complexity, targeting metabolism for therapy is appealing owing to the relative druggability of metabolic enzymes and the multitude of metabolic alterations observed in cancer. *MTAP* biallelic deletion stands out as one of the most prevalent oncogenic events across a diverse range of cancers (10, 11). Previous studies revealed that MTA accumulation resulting from MTAP loss sensitizes cells to short hairpin RNA–mediated depletion of MAT2A and PRMT5 (10–13). However, PRMT5 inhibitors do not replicate the effects of RNAi-mediated silencing of the target, likely due to their SAM-uncompetitive mechanism (52, 53), which does not synergize with MTA. Thus, they fail to harness the elevations of MTA observed in *MTAP*-deleted cancers. Another PRMT5 inhibitor of MRTX1719 exhibits synthetic lethality in preclinical models and activity in phase I trial for patients with *MTAP*-null cancer (31), The efficacy remains to be investigated in late-stage trials. While MAT2A inhibition in these cancers represents an alternative strategy, poor pharmacokinetics and cellular adaptation induction blunt their utility.

At the same time, cancer cells generally display specific vulnerabilities to metabolites owing to their high rate of consumption and proliferation. Met dependence in cancer cells is one of such vulnerabilities (46, 54). MR has been shown to be effective against many cancers in vitro and in vivo (46, 54, 55). Upon MR, MAT2A was upregulated to convert Met to SAM to restore the reduced SAM levels. Therefore, targeting MAT2A is a potential therapeutic strategy to suppress tumor growth by diminishing SAM recovery. As mentioned above, devising effective MAT2A inhibitors is challenging, and no MAT2A inhibitors are currently available in clinical practice. On the other hand, SAM depletion–induced MAT2A upregulation is dependent on METTL16-mediated N6-methyladenosine in the 3′ UTR of MAT2A (32–34); therefore, targeting METTL16 is a potentially feasible treatment strategy to inhibit MAT2A. We have revealed that DNA damage induces METTL16 conformational change and autoinhibition. Indeed, *MTAP* deletion confers vulnerability to genotoxic agents in our analysis, in which PARPi showed the most remarkable effect. PARPi also exhibited a better combination effect with *MTAP* deletion compared with PRMT5 or MAT2A inhibition. Furthermore, our results suggest that MTAPi synergizes with PARPi in suppressing cell proliferation in vitro and tumor growth in vivo. Untargeted metabolite profiling revealed that SAM decreased most markedly upon treatment of MTAPi and olaparib, and this was verified in cellular and mouse models. MR can further strengthen the antitumor efficacy of MTAPi and PARPi by diminishing SAM.

DSBs are regarded as one of the most cytotoxic forms of DNA damage, capable of inducing mutation and initiating either permanent growth arrest or cell death (56, 57). There are two major strategies of nonhomologous end-joining and HR in eukaryotic cells to repair DSBs. Nonhomologous end-joining is a rapid, high-capacity pathway that directly joins the broken ends together (58). By contrast, HR is a high-fidelity and error-free DNA repair mechanism involving a coordinated series of complex steps composed of DNA end resection, RAD51 filament arrangement on the resulting ssDNA to pair homologous sequence, heteroduplex formation, and resolution (59, 60). Upon DNA damage, the MRE11-RAD50-NBS1 complex acts as a sensor of DSBs and is quickly recruited to the damage sites. MRE11 plays a key role in initiating DNA end resection to generate short stretches of ssDNA (61). Methylation within the GAR motif of MRE11 is critical for the capacity of DNA binding as well as exonuclease activity of MRE11 during DNA end resection. Given that SAM is the sole methyl group donor in methylation modification, we speculated that SAM reduction following *MTAP* deficiency combining PARPi and MR can attenuate DNA repair by impairing methylation of MRE11. Indeed, our results validated this hypothesis. Here, we identified a feed-forward loop between DNA repair defect and MR, which is linked by MAT2A and SAM.

BrM is a common and devastating manifestation of breast cancer (1). Although HER2^+^ breast cancer is the most common subtype in breast cancer with BrM, these patients usually have a relative superior survival due to the availability of effective HER2-directed targeted therapies, many of which have demonstrated substantial intracranial activity. Unfortunately, the median overall survival of TNBC with BrM is only 4–6 months owing to the lack of effective systemic therapy with marked intracranial activity (3–5). The limited treatment options for TNBC with BrM have left the door of further drug development for these patients wide open. We have demonstrated that MR enhances the combination effect of PARPi and *MTAP* deficiency/inhibition in tumor growth suppression. It is well established that the brain is an inherent Met-limited environment. Expectedly, we found that *MTAP* deficiency or inhibition confers susceptibility to PARPi in brain metastatic TNBC.

Taken together, our data indicated that PARPi confers MAT2A inhibition by attenuating METTL16-mediated mRNA splicing. *MTAP* deficiency/inhibition enables susceptibility to PARPi by reducing SAM levels. Depletion of SAM, in turn, impairs PARPi-induced DNA damage repair by attenuating end resection and MRE11 recruitment to DSBs sites. More importantly, we revealed that *MTAP* deficiency/inhibition combined with low doses of PARPi substantially kills brain metastatic TNBC owing to the inherent Met-limited environment. Overall, our results offer an encouraging therapeutic scheme for TNBC.

## Methods

### Sex as a biologic variable.

All animal experiments used only female mice because the vast majority of breast cancers occur in women.

### Cell lines.

All cell lines were obtained from the sources indicated in the Supplemental Table 4 and cultured in a humidified 5% CO_2_ incubator at 37°C. HEK293T cells were cultured in DMEM medium. HCC70 cells were cultured in RPMI-1640 medium modified to contain 2 mM L-glutamine and 4,500 mg/L glucose supplemented with 10% FBS. BT549 cells were cultured in RPMI-1640 medium modified to contain 2 mM L-glutamine, 4,500 mg/L glucose supplemented with 0.01 mg/mL insulin, and 10% FBS. MCF12A and HMEC cells were cultured in Mammary Epithelial Cell Growth Medium (Lonza; catalog CC-3150). All cell lines were utilized before passage 20, and testing for mycoplasma contamination was performed on a regular basis.

### Chemicals.

All the chemicals used in this study are listed in Supplemental Table 5.

### Lentivirus production and infection.

Lentiviruses were packaged in HEK293T cells in which indicated constructs, pMD2.G, and pSPAX2, were cotransfected using TransIT-X2 transfection reagent (MIRUS Bio). Supernatants were collected at 48 and 72 hours after transfection. Harvested media with 8 μg/mL polybrene were added to infect cells for further experiments.

### Generation of CRISPR/cas9-KO cell lines.

As previously described (35), *MTAP* sgRNA2 (5′-TTGAGGGAGGAGATTCAGCC-3′) was cloned into pX458-Ef1a-dCas9-KRAB-MECP2-H2B-mCherry and *MTAP* sgRNA1 (5′-GCCTGGTAGTTGACCTTTGA-3′), *METTL16* sgRNA1 (5′-GCATGCAAGAAATAGATACA-3′), and *METTL16* sgRNA2 (5′-CATGTTCAGATAAATCTGAA-3′) were cloned into pSpCas9(BB)-2A-GFP (PX458) plasmid according to the standard protocol. The sgRNA/Cas9 expression constructs were transfected into indicated cells. Twenty-four hours after transfection, the cells were enriched by fluorescence-based sorting using a FACS Aria SORP (BD Biosciences) and transferred into 96-well plates at approximately 1 cell per well. For *METTL16* KO, GFP expression cells were sorted; for *MTAP* KO, both mCherry and GFP expression cells were sorted. Single cells were grown for approximately 6 weeks, and colonies were screened for KOs by genomic sequencing and Western blotting.

### Cell viability.

Cell viability was measured by Cell Titer 96 aqueous nonradioactive cell viability assay (MTS assay, Promega). Cells were seeded onto 96-well culture plates at a density of 4,000 cells/well. After treatment with a compound or a combination of compounds for 72 hours, cells were analyzed for cell viability according to the manufacturer’s instructions.

### Plate colony formation assay.

Cells (500–1,000) were seeded in triplicate in each well of 6-well plates and then treated as indicated. After incubation for 14 days at 37°C, colonies were stained with 5% GIEMSA (51811-82-6, MilliporeSigma) and counted. The survival of untreated cells was normalized to 100%.

### RT-qPCR.

RNA was isolated using the RNeasy Kit (Qiagen), and cDNA was made using the SuperScript III first-strand cDNA synthesis Kit (Thermo Fisher Scientific). Reverse transcriptase quantitative PCR (RT-qPCR) was performed using the Power SYBR Green PCR Master Mix (Thermo Fisher Scientific) and the 7500 real-time PCR system (Applied Biosystems). The primers for RT-qPCR used in this study are collected in Supplemental Table 6.

### Protein extraction and Western blot assay.

Protein extraction commenced by washing the cell pellet with PBS, followed by lysis in RIPA buffer (R0278, MilliporeSigma) supplemented with 5 mM EDTA, 1× Halt phosphatase inhibitor cocktail (78420, Thermo Fisher Scientific), and 1× Halt protease inhibitor cocktail (78429, Thermo Fisher Scientific) on ice for 20 minutes. Subsequently, centrifugation at 12,000*g* at 4°C for 15 minutes separated the supernatant, from which protein concentration was determined using the BCA method. Western blots were performed according to standard laboratory protocols. Blots were visualized and quantified using the ChemiDoc MP Imaging System (Bio-Rad) and the ImageJ software (NIH). Antibodies used in this study are listed in the Supplemental Table 7.

### Soluble and chromatin fractions extraction.

Cells subjected to specified treatments were lysed using NETN buffer supplemented with protease and phosphatase cocktail inhibitors (Thermo Fisher) for 15 minutes on ice, followed by centrifugation at 12,000*g* for 10 minutes. The resulting supernatants constituted the soluble fractions. Pellets were subsequently resuspended in Buffer B (containing Tris-HCl [pH 7.5] 50 mM, NaCl 150 mM, NP-40 1.0%, SDS 0.1%, Na-deoxycholic acid 0.1%, glycerol 5%, DNase 20 μg/mg, and NEM 10 mM), subjected to sonication for approximately 20 seconds, and then centrifuged for 5 minutes at 12,000*g*. The supernatant from this centrifugation step was transferred to a new tube, constituting the chromatin fractions.

### Coimmunoprecipitation.

Protein extraction was described as above. The supernatant was subjected to indicated antibodies with protein A/G-Sepharose beads (Amersham Biosciences) and rotated overnight at 4°C. Beads were washed with NETN buffer 5 times, and samples were boiled with 50 μL protein loading buffer and immunoblotted with indicated antibodies.

### HR reporter assay.

As previously described (62), the indicated cells were transfected with DR-GFP, I-SceI, and mCherry. Forty-eight hours later, cells were harvested and fixed, the percentage of GFP/mCherry^+^ cells was counted by flow cytometry (FACS). Results were normalized to those of the control group.

### Immunofluorescence staining.

Cells cultured on coverslips were treated with olaparib, followed by the indicated recovery time. Subsequently, depending on the foci to be stained, cells underwent sequential steps: being washed with PBS; preextracted with a solution containing 20 mM HEPES pH 7.4, 50 mM NaCl, 3 mM MgCl_2_, 300 mM sucrose, and 0.5% NP-40 for 2 minutes on ice; incubated in 3% paraformaldehyde for 15 minutes; and permeabilized in 0.25% Triton X-100 solution for 5 minutes at room temperature. For RPA32 staining, fixation was done at –20°C using a 1:1 mixture of acetone/methanol. After PBS washes, cells were fixed again with 3% paraformaldehyde for 15 minutes. Subsequently, samples were incubated overnight at 4°C with the primary antibody, followed by 3 washes and a 1-hour incubation with the secondary antibody at room temperature. DAPI staining was performed to visualize nuclear DNA. Finally, coverslips were mounted onto glass slides with ProLong Gold antifade solution (P36934, Thermo Fisher Scientific) and observed using a Nikon Eclipse 80i fluorescence microscope.

### Single-cell gel electrophoresis/comet assay.

The specified cells were seeded at a density of 2 × 10^5^ cells per well in 6-well plates and subjected to analysis using the Comet single-cell gel electrophoresis assay kit (Trevigen, 4250-050-K) following the manufacturer’s protocol. Initially, cells were encapsulated in low melting agarose on a glass slide and incubated in lysis buffer for 1 hour at 4°C. Following this, slides were treated with neutral electrophoresis buffer for 60 minutes to facilitate DNA unwinding. Electrophoresis was then conducted for 30 minutes at 300 mA. Subsequently, slides were immersed in 70% ethanol for 5 minutes, air dried, and stained with SYBR. For quantification, comets on each gel were visualized using a Nikon Eclipse 80i fluorescence microscope, with tail length and tail moment serving as metrics for assessing DNA damage.

### Untargeted metabolite profiling by LC-MS.

Untargeted metabolites LC-MS was performed as previously described (13). Briefly, approximately 1 × 10^7^ cells underwent two 1-minute homogenization cycles at maximum speed using a Retschmill (MM 301, Retsch). Metabolites were then extracted in 1 mL precooled methanol/methyl-tert-butyl-ether/water (1:3:1) with 10 minutes of shaking at 4°C, followed by 10 minutes of incubation in an ice-cooled ultrasonication bath. The homogenate received 650 mL UPLC-grade methanol/water (1:3), was vortexed, and was spun for 5 minutes at 4°C, resulting in phase separation with polar and semipolar metabolites in the lower aqueous phase. The separated phase was isolated, dried in a SpeedVac, and stored at –80°C for subsequent LC-MS analysis. LC-MS data were acquired using a Waters Acquity UPLC system coupled with an Exactive mass spectrometer (Thermo Fisher). Chromatograms were analyzed and processed with REFINER MS 10.0 (GeneData), extracting molecular masses, retention times, and associated peak intensities from the.raw files. Chemical noise subtraction was automated, and chromatogram alignments utilized pairwise alignment-based trees with *m/z* windows of 5 points and retention-time windows of 5 scans within a sliding frame of 200 scans. Further data processing included isotope clustering, adduct detection, and library search, generating data matrices with peak ID, retention time, and peak intensities. Day normalization and sample median normalization were performed on the resulting data matrices for subsequent analysis.

### SAM and SAH measurement.

Measurement of SAM and SAH was performed using ultra-high-performance liquid chromatography equipped with tandem mass spectrometry, TQD (UPLC-MS/MS; Waters) based on a previous report (63). Separation was achieved using an ACQUITY UPLC BEH C18 column. Briefly, cells were lysed using 3 cycles of freeze/thaw in 50% methanol. Samples were deproteinized using 33% acetonitrile and evaporated completely. Pellets were dissolved in 10 mM HCl, followed by filtration using 0.22 μm polyvinylidene fluoride filter (Millipore) and diluted with equal volumes of 50 mM Tris-HCl (pH 8.8) with 100 μM dithiothreitol (DTT) for SAM and SAH. Each sample was injected, and concentrations were calculated based on the standard curve obtained from serial dilution of the standard solution.

### Mouse studies.

For xenograft assays, 2 × 10^6^ of the indicated cells were injected subcutaneously into the flanks of 6- to 8-week-old nu/nu mice (The Jackson Laboratory). For the PDX experiments, 1 × 10^6^ of TNBC PDX cells were injected subcutaneously into 6-week-old nu/nu mice (The Jackson Laboratory). One week after injection, tumors were measured using calipers. Tumor volumes were calculated using the formula: 0.5 × length × width^2^. When tumors reached approximately 100–200 mm^3^, the mice were randomly allocated to treatment groups. Mice were treated as described in the figure legends and monitored for tumor growth and overall health.

Intracardiac injection to induce brain metastases was performed as described previously (49, 64). Briefly, 1 × 10^5^ cells (in 100 μL PBS) were injected into the left cardiac ventricle of female 5- to 6-week-old nu/nu mice (The Jackson Laboratory). Mice were then immediately injected with D-luciferin (150 mg/kg) and subjected to BLI to ensure systemic dissemination of tumor cells. Metastatic burden was measured weekly after injection using BLI for up to 16 weeks. BLI images were analyzed using Living Image Software v.2.50.

For intracranial tumor mouse models, the specified cells were intracranially injected at coordinates 1 mm anterior and 2 mm lateral of the left hemisphere relative to the bregma, at a depth of 4 mm followed by 7–10 days of tumor establishment. Tumor burden was monitored by the BLI. Tumor signal was detected twice per week.

### TUNEL staining.

Harvested tumors were fixed with formalin and embedded in paraffin. 5-μm sections were cut with a paraffin slicing machine (Leica Mikrosysteme Vertrieb GmbH). TUNEL was performed using a One-Step TUNEL Apoptosis Assay Kit (C1090, Beyotime). Following the TUNEL reaction, the slides were treated with DAPI (F6057, Sigma) according to the manufacturer’s instructions. Images were captured using a confocal microscope. The number of TUNEL^+^ cells was calculated in 6 random fields per slide using ImageJ software (NIH).

### Immunohistochemistry.

Immunohistochemistry staining of MTAP (1:200) was performed using an IHC Select HRP/DAB kit (Millipore, DAB150) according to the manufacturer’s instructions.

### Analysis of drug sensitivity in breast cancer.

Public breast cancer data were downloaded from cBioPortal (https://www.cbioportal.org/). Homozygous deletion of *MTAP* was analyzed by copy number variant. Based on the GDSC data, the *calcPhenotype* algorithm from oncoPredict (R package) (29) was used to evaluate drug IC_50_ values for each sample in TCGA exhibiting WT or homozygous deletion of *MTAP*.

### Statistics.

For quantitative measurements, data are represented as mean ± SD. Statistical significance was determined by 1-way ANOVA, 2-way ANOVA, or unpaired 2-tailed *t* test to compare 2 groups of samples. Kaplan-Meier plot and log-rank (Mantel-Cox) test were used to assess survival curves. Correlation analysis was performed by calculating Pearson’s correlation coefficient. The exact *P* values are shown in figures, and those less than or equal to 0.05 are considered significant. Data were graphed and analyzed using GraphPad Prism v.8. Densitometry analysis of the bands from Western blot was performed with GelAnalyzer (http://www.gelanalyzer.com/?i=1).

### Study approval.

Human TNBC specimen collection was approved by the institutional review boards of Union Hospital, Tongji Medical College, and Huazhong University of Science and Technology, and written informed consent was obtained from all patients for the use of their samples. All animal experiments were approved by Mayo Clinic’s Institutional Animal Care and Use Committee. The Mayo Clinic’s Institutional Animal Care and Use Committee is accredited by the Association for Assessment and Accreditation of Laboratory Animal Care International, and animals were maintained in accordance with the *Guide for the Care and Use of Laboratory Animals*, 8th edition (National Academies Press, 2011).

### Data availability.

The *MTAP* deletion in breast cancer and drug sensitivity data were downloaded from cBioPortal (https://www.cbioportal.org/) and GDSC (https://www.cancerrxgene.org), respectively. Mass spectrometry untargeted metabolite data are provided herein (Supplemental Table 2).

Any additional information required to reanalyze the data reported in this paper is available from the corresponding author upon request. Values for all data points in graphs are reported in the Supporting Data Values file.

## Author contributions

XZ and FZ conceived of and designed the study. XT, YZ, WY, JH, QJ, SZ, and ZW conducted the experiments. YH and LZ analyzed and interpreted the data. LZ and RMW shared LC-MS protocols and experimental insights. XZ, FZ, KT, LW, and ZL wrote and reviewed the manuscript. KT, LW, and ZL supervised the study. The assignment of XZ and FZ as equal co–first authors is solely based on their relative contributions toward data generation, initiation, and completion of the studies. XZ is the first of the two equal first authors as he carried out a major part of the initial experiments.

## Supplementary Material

Supplemental data

Unedited blot and gel images

Supplemental tables 1-7

Supporting data values

## Version 1 07/01/2025

Electronic publication
