# Supplementary material for: Targeting MTAP increases PARP inhibitor susceptibility in triple-negative breast cancer through a feed-forward loop
Source: J Clin Invest. 2025 Jul 1;135(13):e188120. doi: 10.1172/JCI188120 (PMC12208554; doi:10.1172/JCI188120)
Supplement: Supplemental data [file jci-135-188120-s029.pdf]

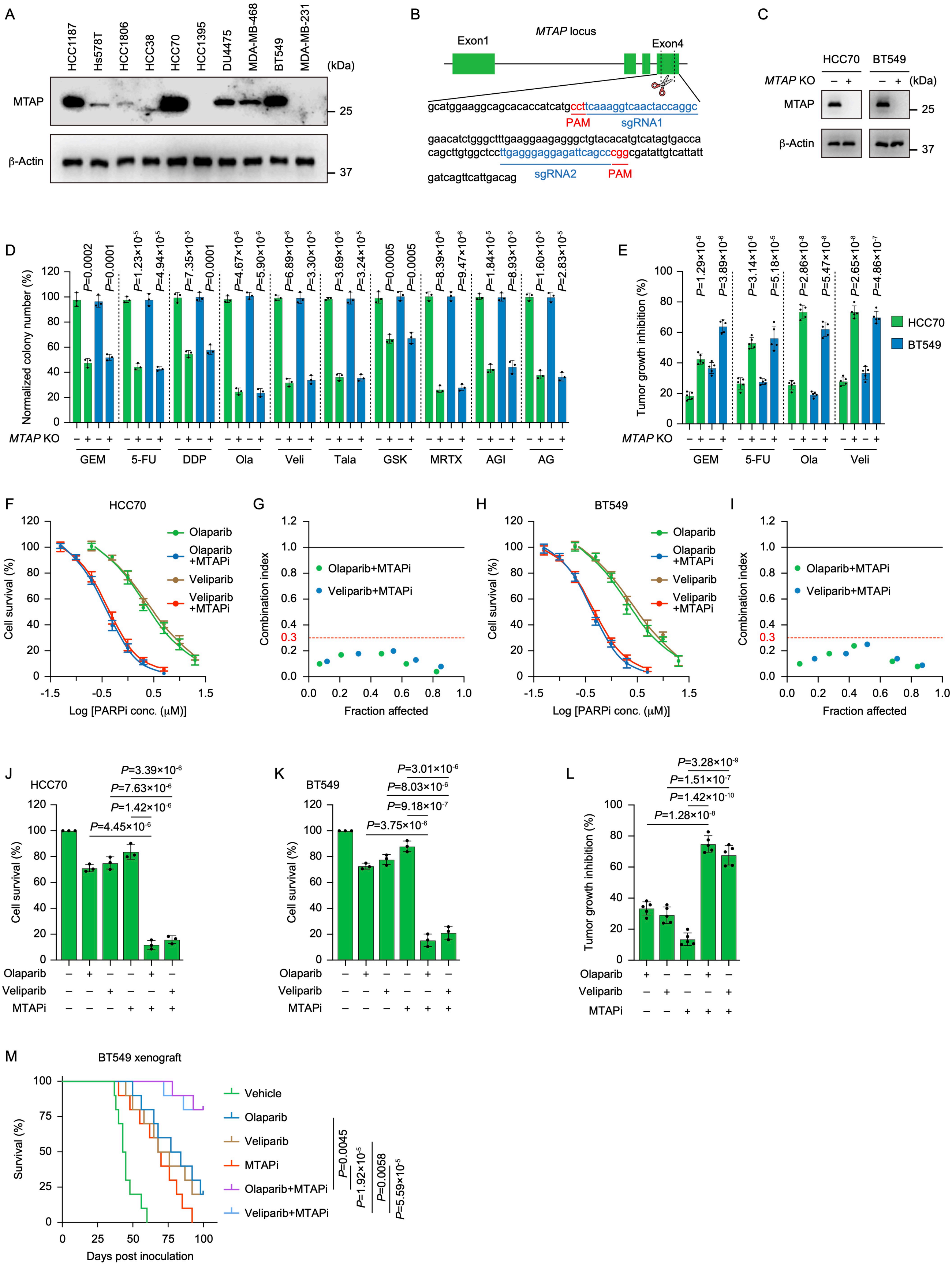

A
HCC1187
Hs578T
HCC1806
HCC38
HCC70
HCC1395
DU4475
MDA-MB-468
BT549
MDA-MB-231
(kDa)
MTAP
25
β-Actin
37
B
MTAP locus
Exon1
Exon4
gcatggaaggcagcacaccatcatgcctcaaaggtcaactaccaggc
PAM
sgRNA1
gaacatctgggctttgaaggaagagggctgtacacatgtcatagtgacca
cagcttgtggctccttgagggaggagattcagcccggcgatattgtcattatt
gatcagttcattgacag
sgRNA2
PAM
C
HCC70
BT549
MTAP KO
(kDa)
MTAP
25
β-Actin
37
D
Normalized colony number (%)
P=0.0002
P=0.0001
P=1.23×10-5
P=4.94×10-5
P=7.35×10-5
P=0.0001
P=4.67×10-6
P=5.90×10-6
P=6.89×10-6
P=3.30×10-5
P=3.69×10-6
P=3.24×10-5
P=0.0005
P=0.0005
P=8.39×10-6
P=9.47×10-6
P=1.84×10-5
P=8.93×10-5
P=1.60×10-5
P=2.83×10-5
MTAP KO
GEM
5-FU
DDP
Ola
Veli
Tala
GSK
MRTX
AGI
AG
E
Tumor growth inhibition (%)
P=1.29×10-6
P=3.89×10-6
P=3.14×10-6
P=5.18×10-5
P=2.86×10-8
P=5.47×10-8
P=2.65×10-8
P=4.86×10-7
HCC70
BT549
MTAP KO
GEM
5-FU
Ola
Veli
F
HCC70
Cell survival (%)
Log [PARPi conc. (μM)]
Olaparib
Olaparib +MTAPi
Veliparib
Veliparib +MTAPi
G
Combination index
Fraction affected
Olaparib+MTAPi
Veliparib+MTAPi
H
BT549
Cell survival (%)
Log [PARPi conc. (μM)]
Olaparib
Olaparib +MTAPi
Veliparib
Veliparib +MTAPi
I
Combination index
Fraction affected
Olaparib+MTAPi
Veliparib+MTAPi
J
HCC70
Cell survival (%)
P=3.39×10-6
P=7.63×10-6
P=1.42×10-6
P=4.45×10-6
Olaparib
Veliparib
MTAPi
K
BT549
Cell survival (%)
P=3.01×10-6
P=8.03×10-6
P=9.18×10-7
P=3.75×10-6
Olaparib
Veliparib
MTAPi
L
Tumor growth inhibition (%)
P=3.28×10-9
P=1.51×10-7
P=1.42×10-10
P=1.28×10-8
Olaparib
Veliparib
MTAPi
M
BT549 xenograft
Survival (%)
Days post inoculation
Vehicle
Olaparib
Veliparib
MTAPi
Olaparib+MTAPi
Veliparib+MTAPi
P=0.0045
P=1.92×10-5
P=0.0058
P=5.59×10-5

Supplemental Figure 1. Targeting MTAP combined with PARPi effectively kill tumor cells expressing MTAP. (**A**) Representative western blot of MTAP in diverse TNBC cell lines. (**B**) Schematic representation of paired sgRNAs designed for targeting *MTAP* exon 4. (**C**) Representative western blots of MTAP in HCC70 and BT549 cells with or without *MTAP* knock out. (**D** and **E**) Cell survival (**D**) and tumor growth inhibition (**E**) analysis of HCC70 and BT549 cells with WT or deleted *MTAP* treated with the indicated DNA damage-inducing agents. GEM, gemcitabine; DDP, cisplatin; Ola, olaparib; Veli, veliparib; Tala, talazoparib; GSK, GSK3326595; MRTX, MRTX1719; AGI, AGI-24512; AG, AG-270. (**F**-**I**) MTS assay and combination index (CI) values of HCC70 (**F** and **G**) and BT549 (**H** and **I**) cells treated with olaparib or veliparib with or without combining with MTAPi (0.1 μM). CI < 0.3 indicates strong synergism. (**J** and **K**) Colony formation assay of HCC70 (**J**) and BT549 (**K**) cells treated with olaparib (0.2 μM), veliparib (0.2 μM), or MTAPi (0.1 μM), or the indicated combination. (**L** and **M**) Tumor growth inhibition (**L**) and Kaplan-Meier survival curves (**M**) of BT549 xenograft tumor models treated with olaparib (50 mg/kg), veliparib (50 mg/kg), or MTAPi (10 mg/kg), or the indicated combination. (**A** and **C**) Experiment was repeated 3 times, and representative blots are presented. (**D**, **F**, **H**, **J**, and **K**) Data are shown as the mean ± SD from 3 independent experiments. (**E** and **L**) Data are shown as the mean ± SD from one representative experiment of 5 mice per group, $n = 10$ mice per group in **M**. P values are indicated. Significance determined using (**D** and **E**) unpaired t, (**J**-**L**) 1-way ANOVA, or (**M**) log-rank (Mantel-Cox) test.

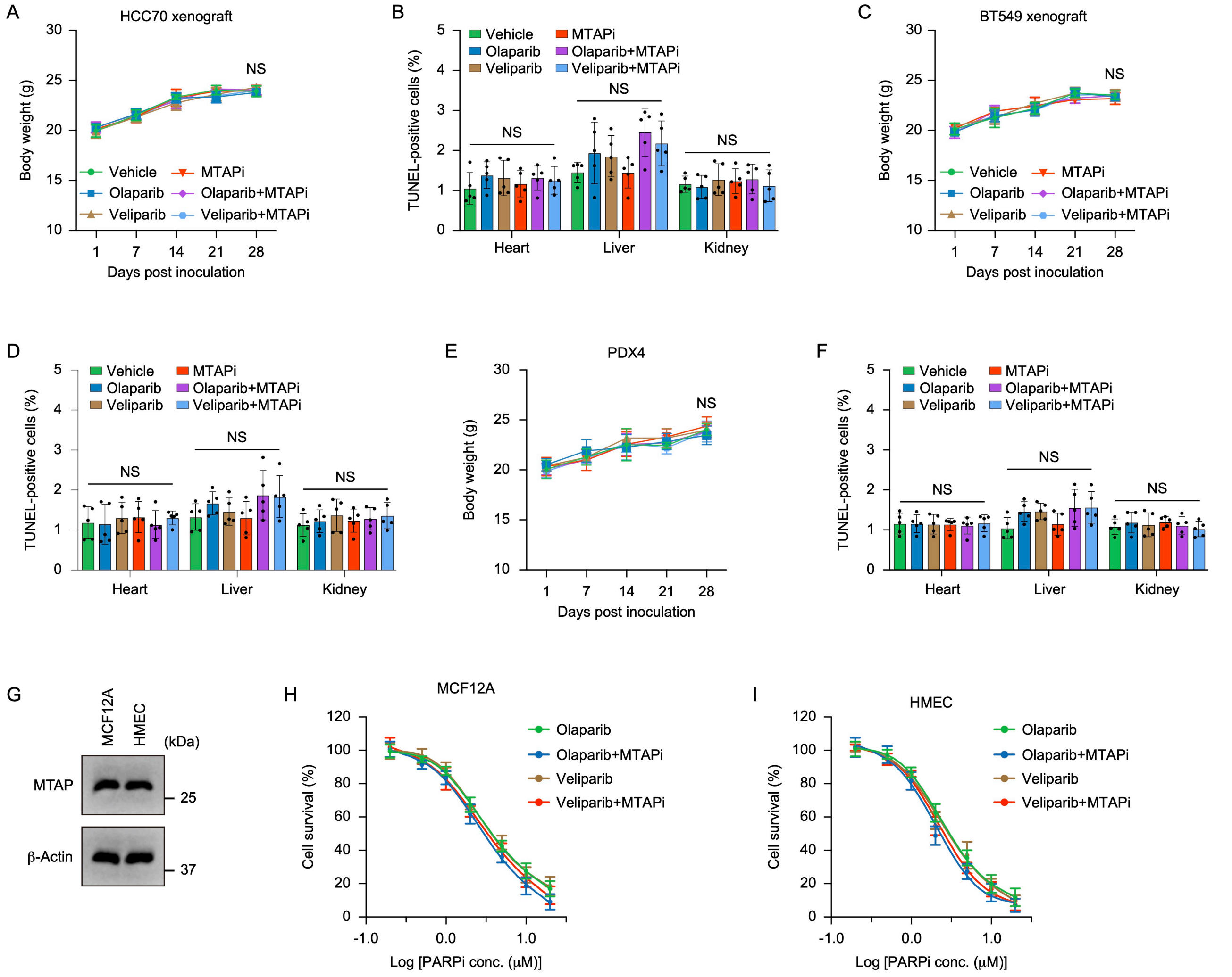

Supplemental Figure 2. Combination of PARPi and MTAPi is well tolerant. (**A-F**) body weight and quantification of TUNEL-positive cells in heart, liver, and kidney of HCC70 (**A** and **B**), BT549 (**C** and **D**), and PDX4 (**E** and **F**) xenograft tumor models treated with olaparib (50 mg/kg), veliparib (50 mg/kg), or MTAPi (10 mg/kg), or the indicated combination. Data are shown as the mean ± SD from one representative experiment of 5 mice per group. (**G**) Representative western blot showing levels of MTAP in the indicated cell lines. Experiment was repeated 3 times, and representative blots are presented. (**H** and **I**) MTS assay of MCF12A (**H**) and HMEC (**I**) cells treated with olaparib or veliparib with or without combining with MTAPi (0.1 μM). Data are shown as the mean ± SD from 3 independent experiments. *P* values are indicated. Significance determined using (**A**, **C**, and **E**) 2-way ANOVA, (**B**, **D**, and **F**) 1-way ANOVA test.

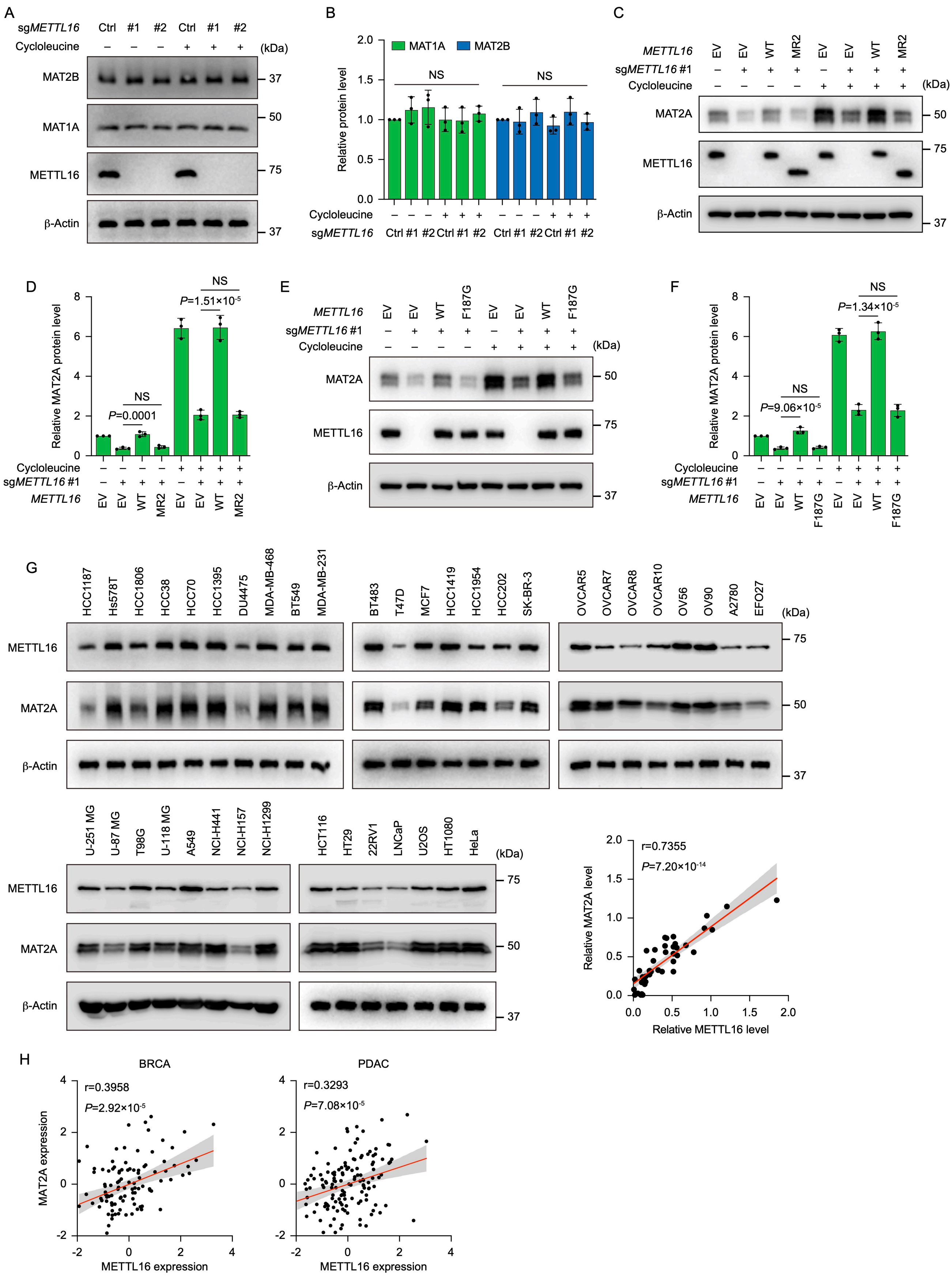

A
sgMETTL16 Ctrl #1 #2 Ctrl #1 #2
Cycloleucine – – – + + +
(kDa)
MAT2B
MAT1A
METTL16
β-Actin
37
50
75
37
B
MAT1A
MAT2B
NS
NS
Relative protein level
2.0
1.5
1.0
0.5
0.0
Cycloleucine
sgMETTL16
Ctrl #1 #2 Ctrl #1 #2 Ctrl #1 #2 Ctrl #1 #2
C
METTL16 EV EV WT MR2 EV EV WT MR2
sgMETTL16 #1 – + + + – + + +
Cycloleucine – – – – + + + +
(kDa)
MAT2A
METTL16
β-Actin
50
75
37
D
Relative MAT2A protein level
8
6
4
2
0
NS
P=1.51×10-5
NS
P=0.0001
Cycloleucine – – – – + + + +
sgMETTL16 #1 – + + + – + + +
METTL16 EV EV WT MR2 EV EV WT MR2
E
METTL16 EV EV WT F187G EV EV WT F187G
sgMETTL16 #1 – + + + – + + +
Cycloleucine – – – – + + + +
(kDa)
MAT2A
METTL16
β-Actin
50
75
37
F
Relative MAT2A protein level
8
6
4
2
0
NS
P=1.34×10-5
NS
P=9.06×10-5
Cycloleucine – – – – + + + +
sgMETTL16 #1 – + + + – + + +
METTL16 EV EV WT F187G EV EV WT F187G
G
HCC1187
Hs578T
HCC1806
HCC38
HCC70
HCC1395
DU4475
MDA-MB-468
BT549
MDA-MB-231
BT483
T47D
MCF7
HCC1419
HCC1954
HCC202
SK-BR-3
OVCAR5
OVCAR7
OVCAR8
OVCAR10
OV56
OV90
A2780
EFO27
(kDa)
METTL16
MAT2A
β-Actin
75
50
37
U-251 MG
U-87 MG
T98G
U-118 MG
A549
NCI-H441
NCI-H157
NCI-H1299
HCT116
HT29
22RV1
LNCaP
U2OS
HT1080
HeLa
(kDa)
METTL16
MAT2A
β-Actin
75
50
37
r=0.7355
P=7.20×10-14
Relative MAT2A level
2.0
1.5
1.0
0.5
0.0
0.0 0.5 1.0 1.5 2.0
Relative METTL16 level
H
BRCA
r=0.3958
P=2.92×10-5
MAT2A expression
4
2
0
-2
-2 0 2 4
METTL16 expression
PDAC
r=0.3293
P=7.08×10-5
4
2
0
-2
-2 0 2 4
METTL16 expression

Supplemental Figure 3. METTL16 positively regulates MAT2A. (**A** and **B**) Representative western blots (**A**) and quantitation (**B**) showing levels of MAT2B, MAT1A, and METTL16 in cells expressing control or *METTL16* sgRNAs with or without cycloleucine (20 mM) treatment. (**C**-**F**) Representative western blots and quantitation showing levels of MAT2A and METTL16 in *METTL16*-null cells re-introduced *METTL16* WT, MR2 (**C** and **D**) or F187G (**E** and **F**) vectors with or without cycloleucine (20 mM) treatment. (**G**) Representative western blots showing METTL16 and MAT2A expression levels and Pearson correlation analysis in diverse cell lines. (**H**) Correlation of METTL16 and MAT2A protein expression in Reverse Phase Protein Array (RPPA) of breast cancer and pancreatic ductal adenocarcinoma. Data are from https://www.cbioportal.org. (**A**, **C**, **E**, and **G**) Experiment was repeated 3 times, and representative blots are presented. (**B**, **D**, and **F**) Data are shown as the mean ± SD from 3 independent experiments. *P* values are indicated. Significance determined using (**B**, **D**, and **F**) 1-way ANOVA, or (**G** and **H**) Pearson correlation test.

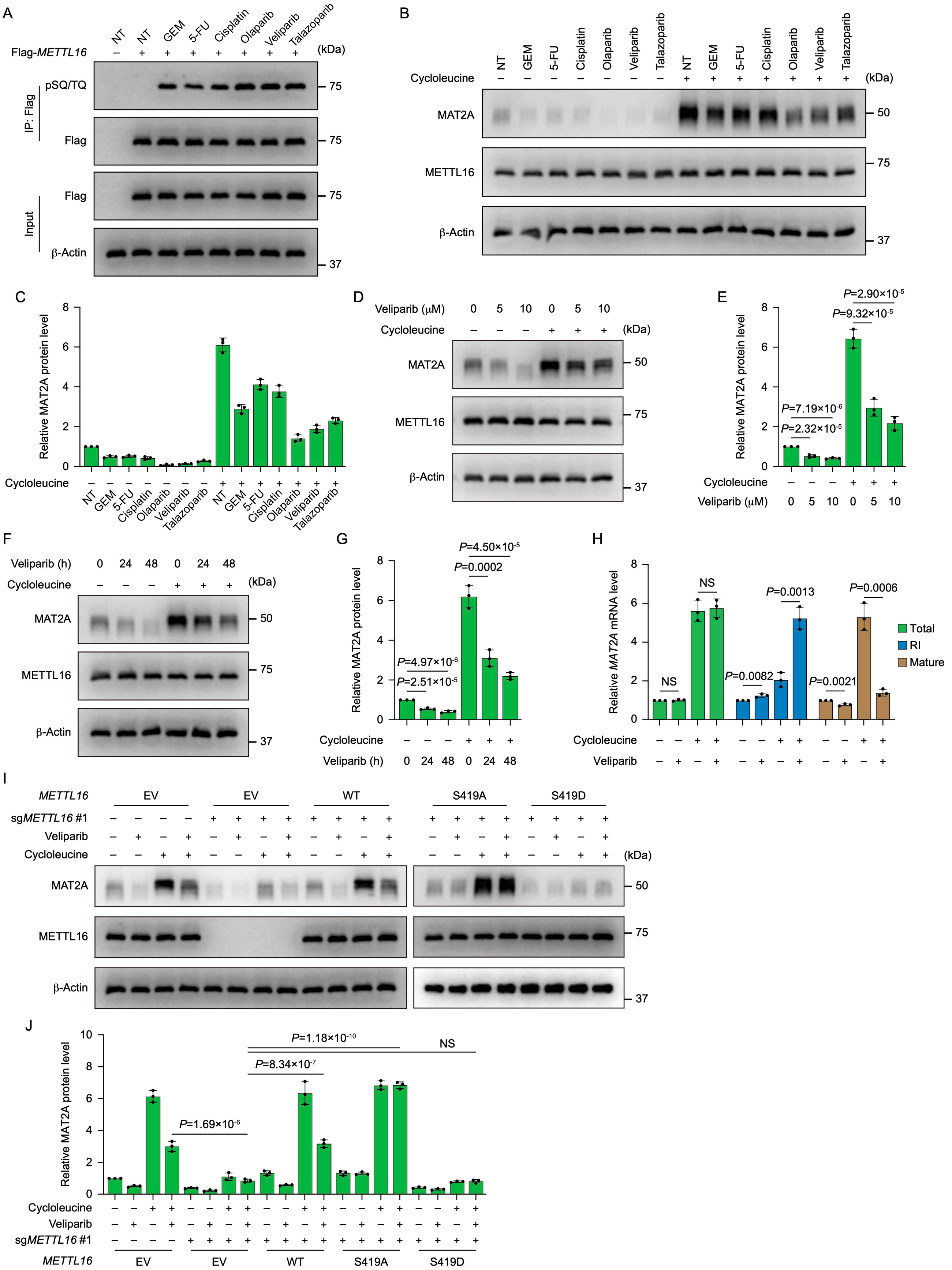

A
NT
NT
GEM
5-FU
Cisplatin
Olaparib
Veliparib
Talazoparib
Flag-METTL16
(kDa)
IP: Flag
pSQ/TQ
Flag
Input
Flag
β-Actin
75
75
75
37
B
Cycloleucine
NT
GEM
5-FU
Cisplatin
Olaparib
Veliparib
Talazoparib
(kDa)
MAT2A
METTL16
β-Actin
50
75
37
C
Relative MAT2A protein level
Cycloleucine
NT
GEM
5-FU
Cisplatin
Olaparib
Veliparib
Talazoparib
D
Veliparib (μM)
0
5
10
Cycloleucine
(kDa)
MAT2A
METTL16
β-Actin
50
75
37
E
Relative MAT2A protein level
P=2.90×10⁻⁵
P=9.32×10⁻⁵
P=7.19×10⁻⁶
P=2.32×10⁻⁵
Cycloleucine
Veliparib (μM)
F
Veliparib (h)
0
24
48
Cycloleucine
(kDa)
MAT2A
METTL16
β-Actin
50
75
37
G
Relative MAT2A protein level
P=4.50×10⁻⁵
P=0.0002
P=4.97×10⁻⁶
P=2.51×10⁻⁵
Cycloleucine
Veliparib (h)
H
Relative MAT2A mRNA level
NS
NS
P=0.0013
P=0.0006
P=0.0082
P=0.0021
Total
RI
Mature
Cycloleucine
Veliparib
I
METTL16
EV
EV
WT
S419A
S419D
sgMETTL16 #1
Veliparib
Cycloleucine
(kDa)
MAT2A
METTL16
β-Actin
50
75
37
J
Relative MAT2A protein level
P=1.18×10⁻¹⁰
NS
P=8.34×10⁻⁷
P=1.69×10⁻⁶
Cycloleucine
Veliparib
sgMETTL16 #1
METTL16
EV
EV
WT
S419A
S419D

Supplemental Figure 4. PARPi attenuates MAT2A expression by suppressing METTL16 facilitated mRNA intron retention. (**A**) Representative western blots showing levels of METTL16 pSQ/TQ with the indicated treatment after transfection with Flag-*METTL16* and immunoprecipitated with Flag. (**B** and **C**) Representative western blots (**B**) and quantitation (**C**) showing levels of MAT2A and METTL16 with the indicated treatment. (**D-G**) Representative western blots and quantitation showing levels of MAT2A and METTL16 in HCC70 cells treated with the indicated dose of veliparib for 24 h (**D** and **E**) or the indicated time period of veliparib (5 μM) (**F** and **G**). (**H**) RT-qPCR analysis of total, intron retention, and mature *MAT2A* mRNA levels in HCC70 cells treated with veliparib (5 μM), or cycloleucine (20 mM), or their combo. (**I** and **J**) Representative western blots (**I**) and quantitation (**J**) showing levels of MAT2A and METTL16 in HCC70 cells stably expressing the indicated vectors treated with veliparib (5 μM), or cycloleucine (20 mM), or their combo. GEM (10 μM), 5-FU (10 μM), cisplatin (10 μM), olaparib (5 μM), veliparib (5 μM), and talazoparib (2 μM) were used in **A** and **B**. (**A**, **B**, **D**, **F**, and **I**) Experiment was repeated 3 times, and representative blots are presented. (**C**, **E**, **G**, **H**, and **J**) Data are shown as the mean ± SD from 3 independent experiments. *P* values are indicated. Significance determined using (**E**, **G**, **H**, and **J**) 1-way ANOVA test. NT, not treatment.

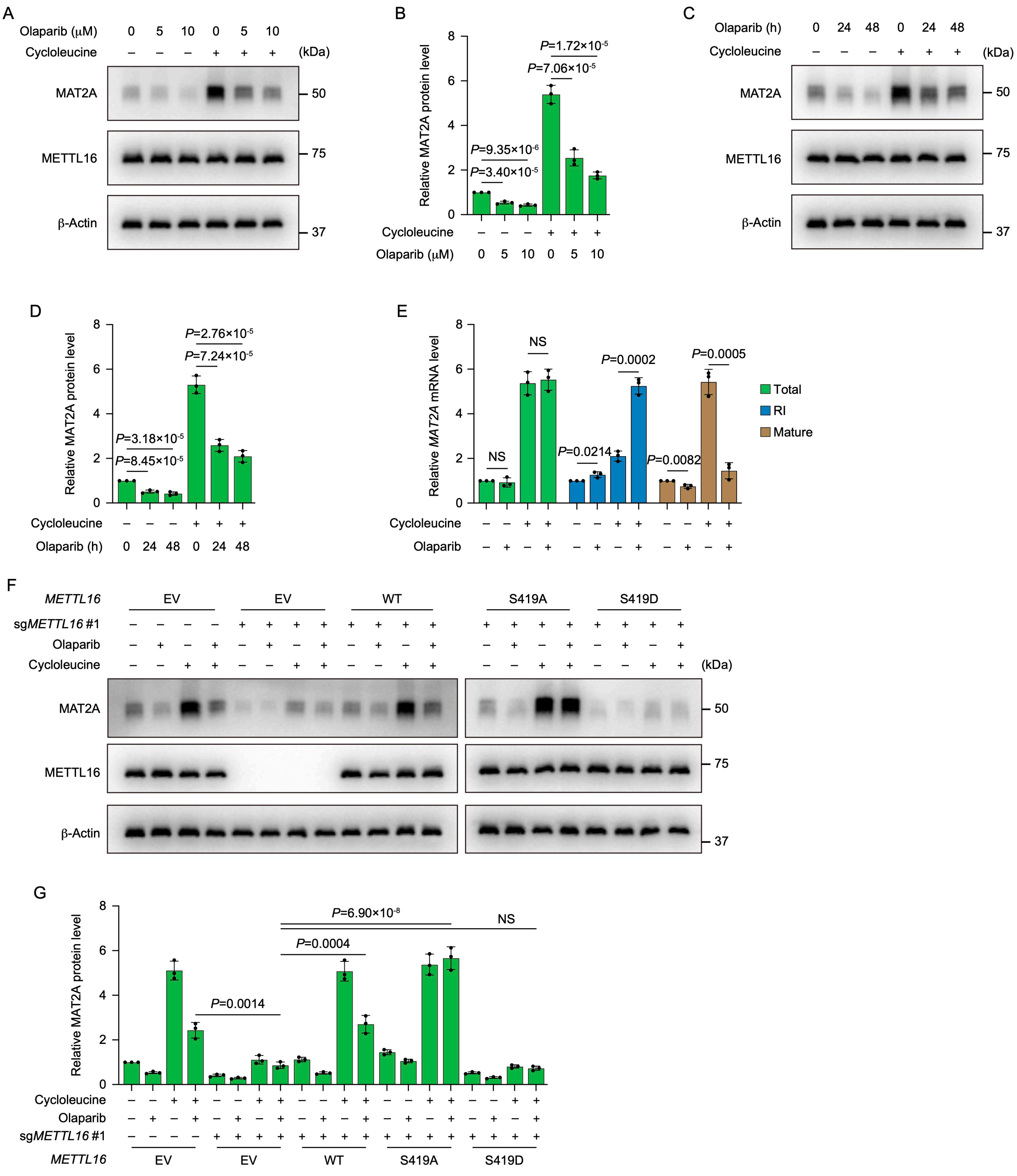

A
Olaparib (μM) 0 5 10 0 5 10
Cycloleucine – – – + + +
(kDa)
MAT2A
50
METTL16
75
β-Actin
37
B
Relative MAT2A protein level
P=1.72×10-5
P=7.06×10-5
P=9.35×10-6
P=3.40×10-5
Cycloleucine – – – + + +
Olaparib (μM) 0 5 10 0 5 10
C
Olaparib (h) 0 24 48 0 24 48
Cycloleucine – – – + + +
(kDa)
MAT2A
50
METTL16
75
β-Actin
37
D
Relative MAT2A protein level
P=2.76×10-5
P=7.24×10-5
P=3.18×10-5
P=8.45×10-5
Cycloleucine – – – + + +
Olaparib (h) 0 24 48 0 24 48
E
Relative MAT2A mRNA level
NS
NS
P=0.0002
P=0.0005
P=0.0214
P=0.0082
Total
RI
Mature
Cycloleucine
Olaparib
F
METTL16 EV EV WT S419A S419D
sgMETTL16 #1
Olaparib
Cycloleucine
(kDa)
MAT2A
50
METTL16
75
β-Actin
37
G
Relative MAT2A protein level
P=6.90×10-8
NS
P=0.0004
P=0.0014
Cycloleucine
Olaparib
sgMETTL16 #1
METTL16 EV EV WT S419A S419D

Supplemental Figure 5. PARPi reduces MAT2A expression via repressing METTL16 mediated mRNA intron retention. (**A-D**) Representative western blots and quantitation showing levels of MAT2A and METTL16 in BT549 cells treated with the indicated dose of olaparib for 24 h (**A** and **B**) or the indicated time period of olaparib (5 μM) (**C** and **D**). (**E**) RT-qPCR analysis of total, intron retention, and mature *MAT2A* mRNA levels in BT549 cells treated with olaparib (5 μM), or cycloleucine (20 mM), or their combo. (**F** and **G**) Representative western blots (**F**) and quantitation (**G**) showing levels of MAT2A and METTL16 in BT549 cells stably expressing the indicated vectors treated with olaparib (5 μM), or cycloleucine (20 mM), or their combo. (**A**, **C**, and **F**) Experiment was repeated 3 times, and representative blots are presented. (**B**, **D**, **E**, and **G**) Data are shown as the mean ± SD from 3 independent experiments. *P* values are indicated. Significance determined using (**B**, **D**, **E**, and **G**) 1-way ANOVA test.

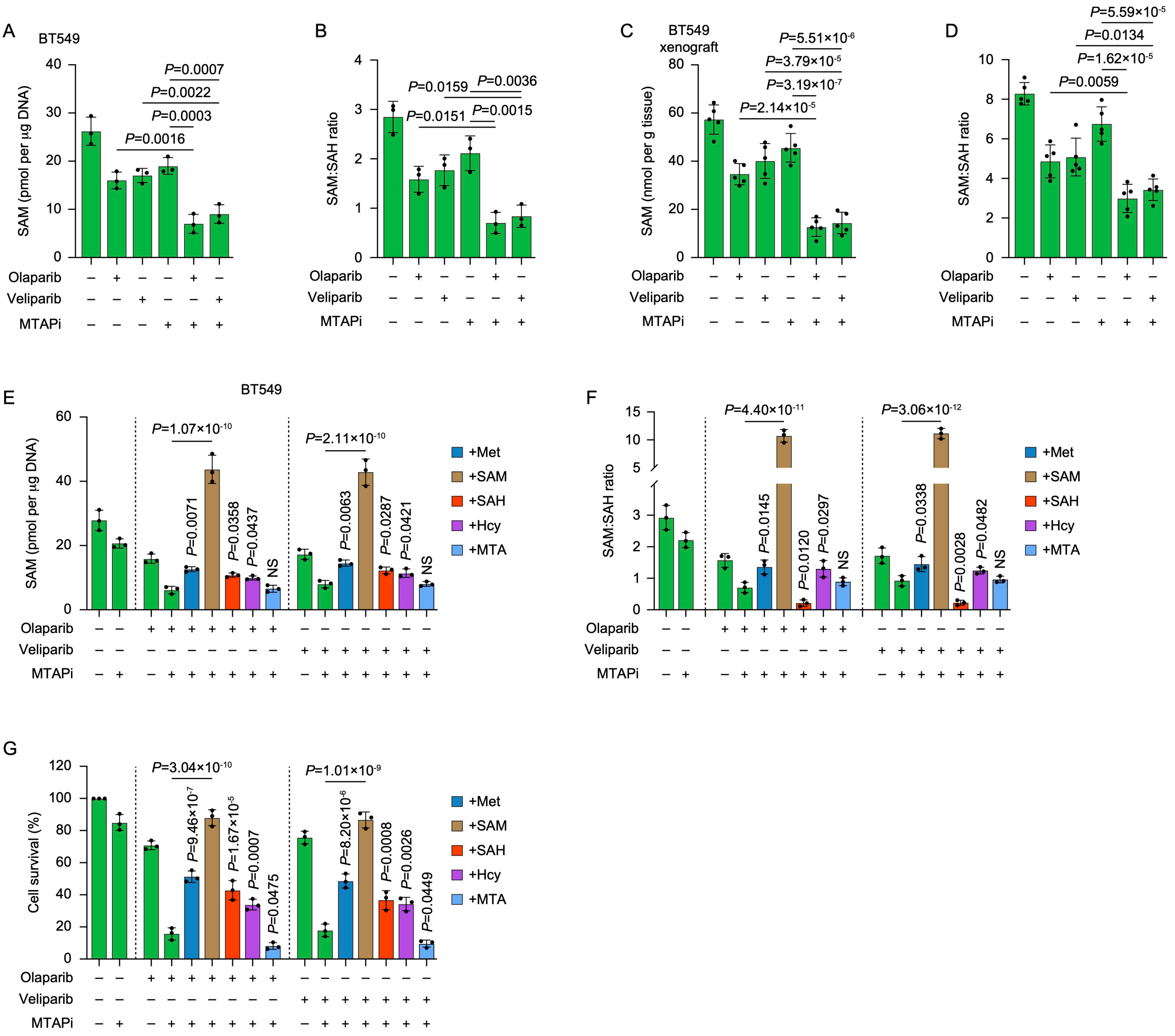

Supplemental Figure 6. Inhibition of MTAP and PARP synergistically eliminates tumor cells by depleting SAM. (**A** and **B**) Intracellular SAM (**A**) and SAM:SAH ratio (**B**) of BT549 cells treated with olaparib (2 μM), veliparib (2 μM), or MTAPi (1 μM), or the indicated combination. (**C** and **D**) Intratumor SAM (**C**) and SAM:SAH ratio (**D**) of BT549 xenograft tumors treated with olaparib (50 mg/kg), veliparib (50 mg/kg), or MTAPi (10 mg/kg), or the indicated combination. Data are shown as the mean ± SD from one representative experiment of 5 mice per group. (**E-G**) Intracellular SAM (**E**), SAM:SAH ratio (**F**), and colony formation assay (**G**) of BT549 cells treated with olaparib, veliparib, or MTAPi, or the indicated combination, and were supplemented with 100 μM Met, SAM, SAH, Hcy, or MTA. (**A**, **B**, and **E-G**) Data are shown as the mean ± SD from 3 independent experiments. *P* values are indicated. Significance determined using (**A-G**) 1-way ANOVA test.

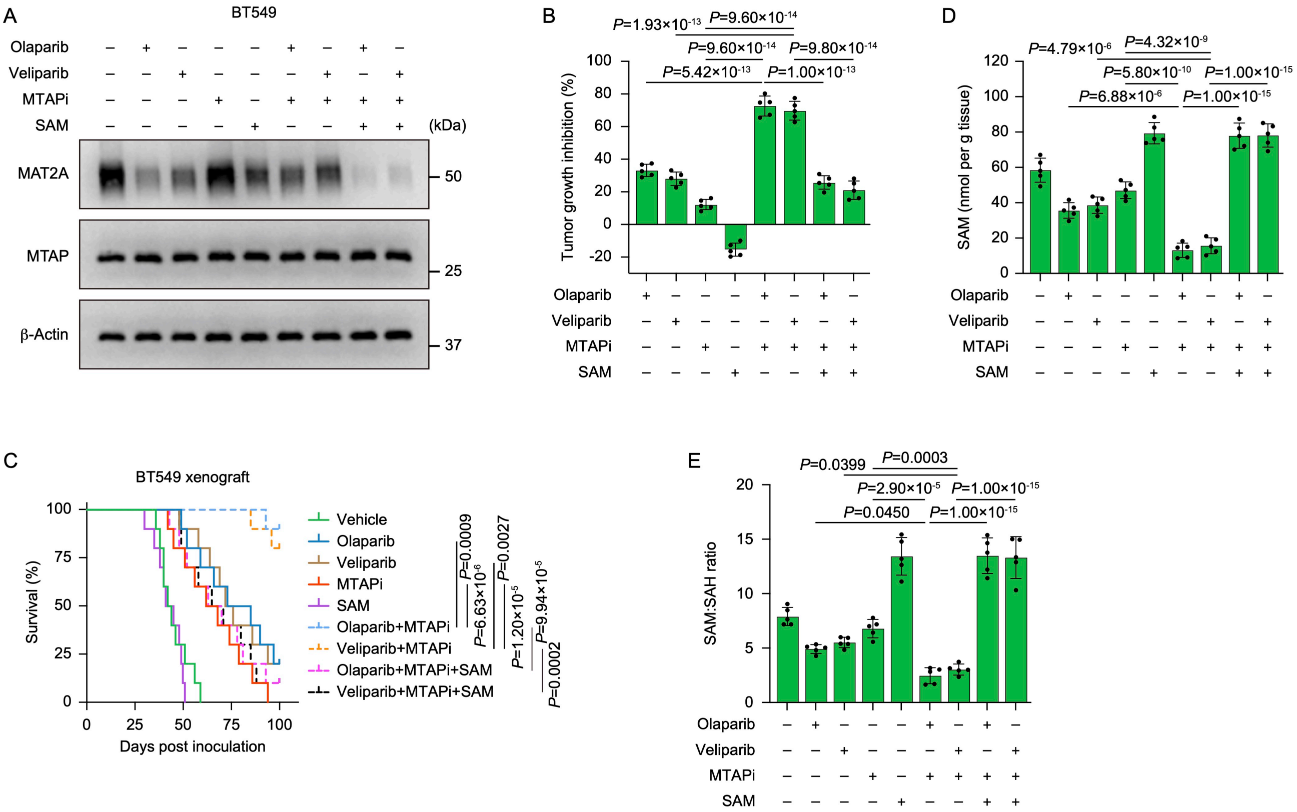

Supplemental Figure 7. Dual inhibition of MTAP and PARP suppresses tumor growth by decreasing SAM. (**A**) Representative western blots showing levels of MAT2A and MTAP in BT549 cells treated with olaparib (2 μM), veliparib (2 μM), MTAPi (1 μM), or SAM (100 μM), or the indicated combination. Experiment was repeated 3 times, and representative blots are presented. (**B**-**E**) Tumor growth inhibition (**B**), Kaplan-Meier survival curves (**C**), intratumor SAM (**D**), and SAM:SAH ratio (**E**) of BT549 xenograft tumors treated with olaparib, veliparib, or MTAPi, or the indicated combination. Olaparib and veliparib were used at 50 mg/kg and MTAPi was used at 10 mg/kg, intraperitoneally, 5 times per week. SAM was administered at 10 mg/kg subcutaneously per day. (**B**, **D**, and **E**) Data are shown as the mean ± SD from one representative experiment of 5 mice per group, n = 10 mice per group in **C**. P values are indicated. Significance determined using (**B**, **D**, and **E**) 1-way ANOVA, or (**C**) log-rank (Mantel-Cox) test.

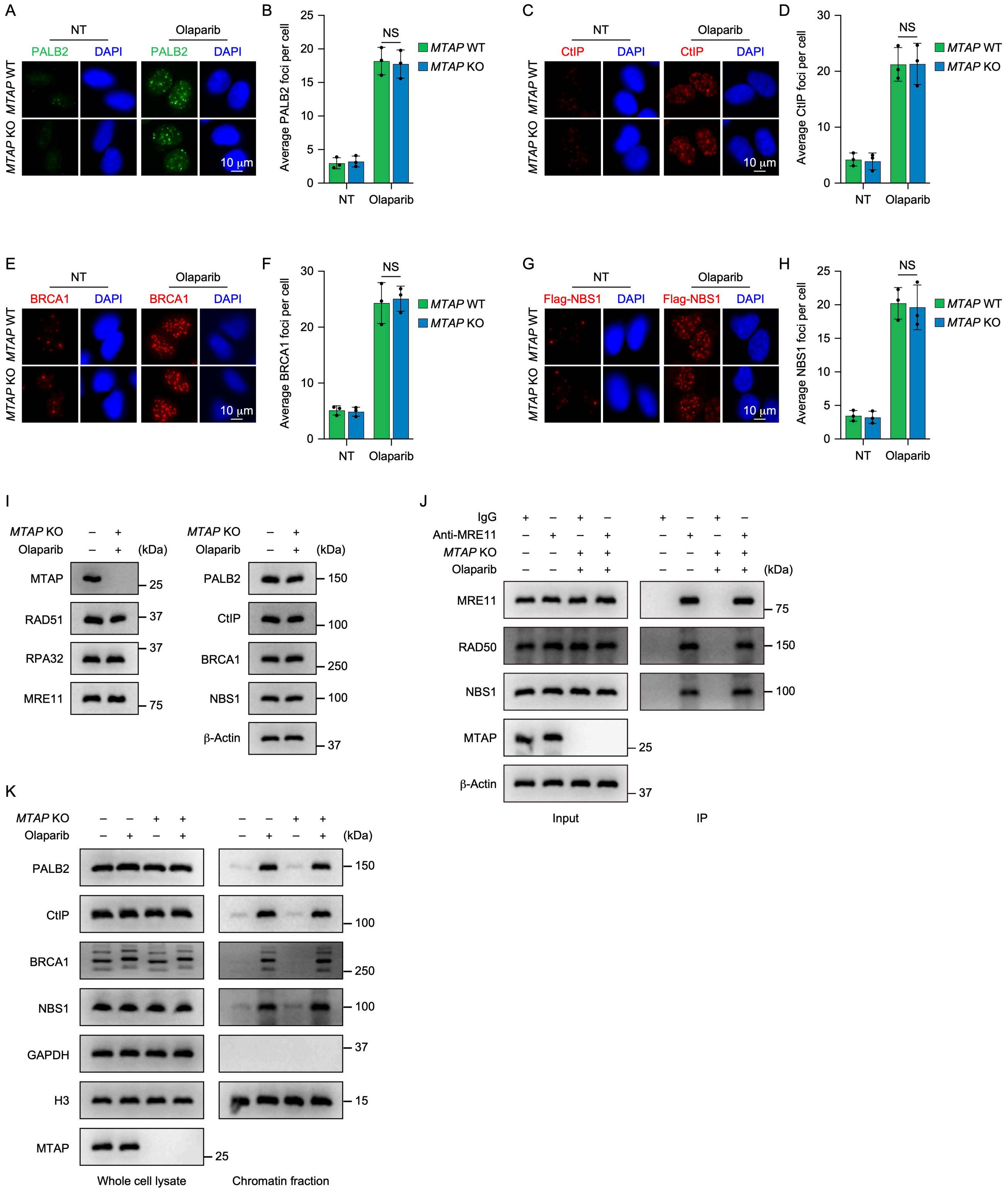

A
NT
Olaparib
PALB2
DAPI
MTAP WT
MTAP KO
10 μm
B
Average PALB2 foci per cell
NS
MTAP WT
MTAP KO
NT
Olaparib
C
CtIP
DAPI
D
Average CtIP foci per cell
E
BRCA1
F
Average BRCA1 foci per cell
G
Flag-NBS1
H
Average NBS1 foci per cell
I
MTAP KO
Olaparib
(kDa)
MTAP
RAD51
RPA32
MRE11
PALB2
CtIP
BRCA1
NBS1
β-Actin
J
IgG
Anti-MRE11
RAD50
Input
IP
K
GAPDH
H3
Whole cell lysate
Chromatin fraction

Supplemental Figure 8. Low SAM blocks DNA repair by inhibiting methylation of MRE11. (**A**-**H**) Representative micrographs and quantitation for PALB2 (**A** and **B**), CtIP (**C** and **D**), BRCA1 (**E** and **F**), or NBS1 (**G** and **H**) foci formation in the indicated cells without treatment, or recovery at 1 h after olaparib (20 μM) treatment. (**I**) Representative western blots immunoblotted with the indicated antibodies in cells with WT or depletion *MTAP* treated with or without olaparib. (**J**) Endogenous IP between MRE11 and RAD50 or NBS1 in cells with WT or depletion *MTAP* treated with or without olaparib (5 μM). (**K**) Representative western blots immunoblotted with the indicated antibodies in whole cell lysate and chromatin fraction of the indicated cells without treatment, or recovery at 1 h after olaparib (20 μM) treatment. (**A**, **C**, **E**, **G**, and **I**-**K**) Experiment was repeated 3 times, and representative micrographs/blots are presented. (**B**, **D**, **F**, and **H**) Data are shown as the mean ± SD from 3 independent experiments. *P* values and scale bars are indicated. Significance determined using (**B**, **D**, **F**, and **H**) unpaired *t* test.

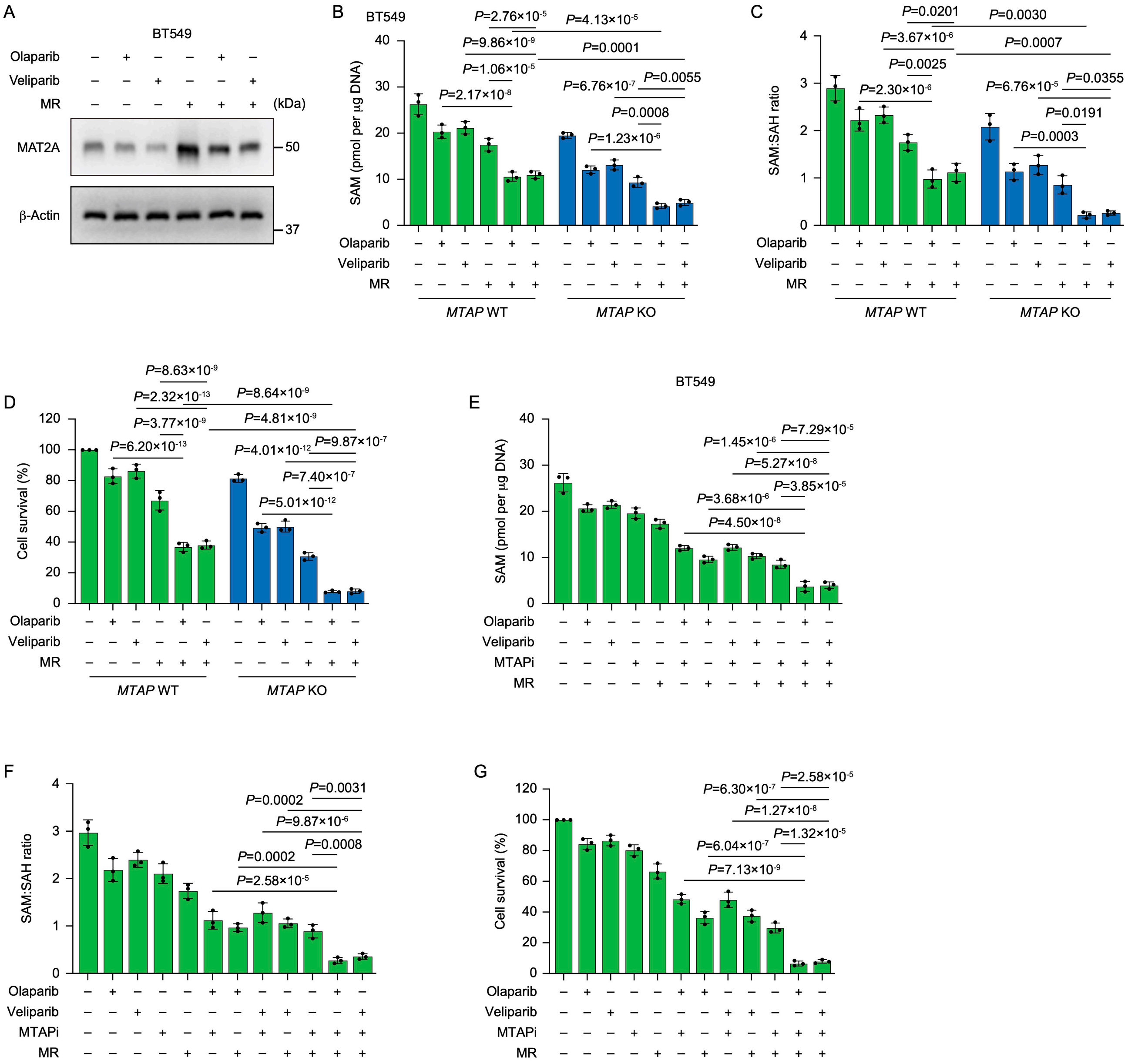

Supplemental Figure 9. MR enhances anti-tumor effect of *MTAP* deficiency/inhibition combining PARPi by promoting SAM reduction. (**A**) Representative western blot showing levels of MAT2A in BT549 cells treated with olaparib (2 µM), veliparib (2 µM), or methionine restriction (MR) (20%), or the indicated combination. Experiment was repeated 3 times, and representative blots are presented. (**B-D**) Intracellular SAM (**B**), SAM:SAH ratio (**C**), and colony formation assay (**D**) of BT549 cells with or without *MTAP* deletion treated with olaparib, veliparib, or MR, or the indicated combination. (**E-G**) Intracellular SAM (**E**), SAM:SAH ratio (**F**), and colony formation assay (**G**) of BT549 cells treated with olaparib, veliparib, MTAPi, or MR, or the indicated combination. (**B-G**) Data are shown as the mean ± SD from 3 independent experiments. *P* values are indicated. Significance determined using (**B-G**) 1-way ANOVA test.

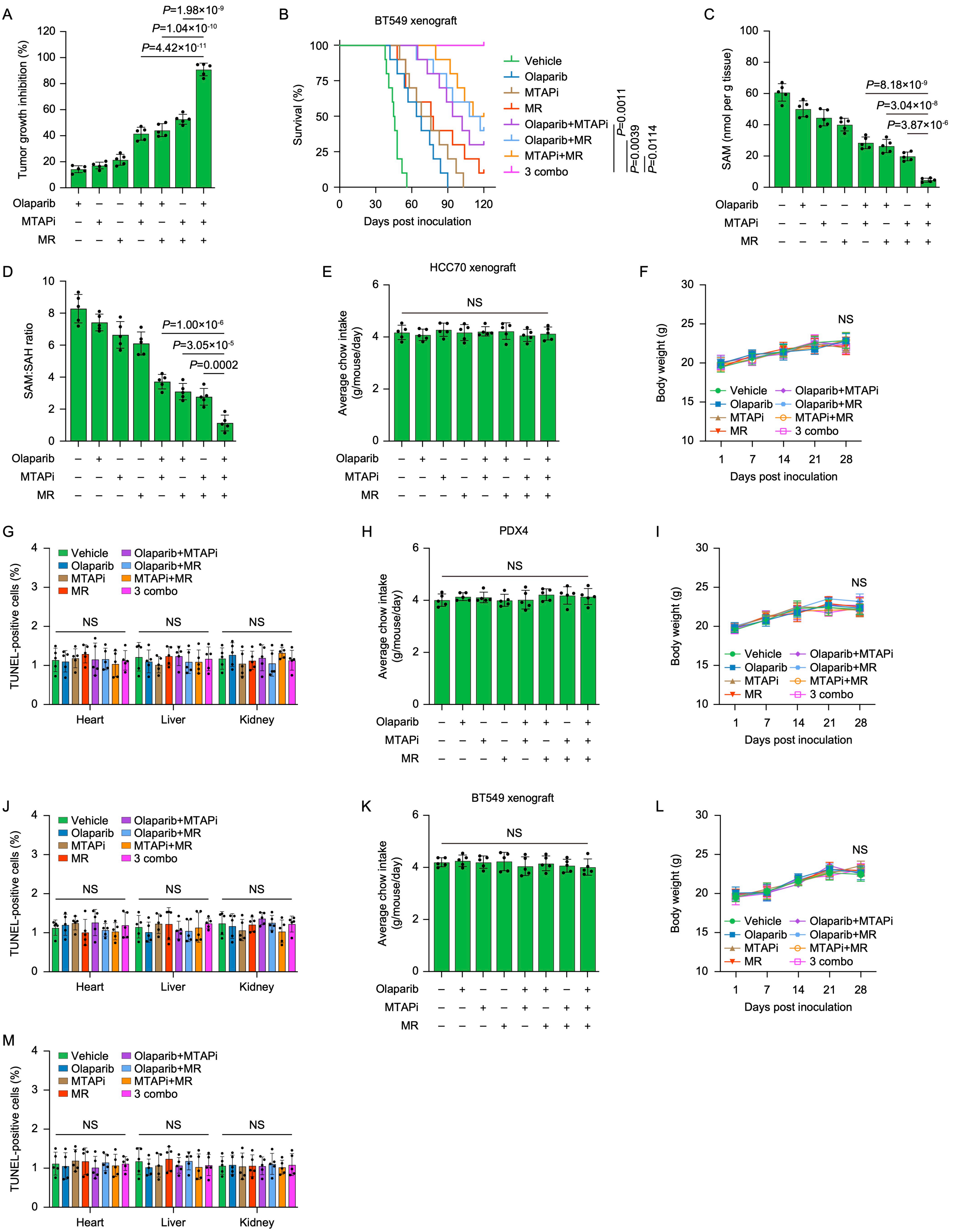
A
P=1.98×10^-9
P=1.04×10^-10
P=4.42×10^-11
Tumor growth inhibition (%)
Olaparib
MTAPi
MR
B
BT549 xenograft
Survival (%)
Days post inoculation
Vehicle
Olaparib
MTAPi
MR
Olaparib+MTAPi
Olaparib+MR
MTAPi+MR
3 combo
P=0.0011
P=0.0039
P=0.0114
C
SAM (nmol per g tissue)
P=8.18×10^-9
P=3.04×10^-8
P=3.87×10^-6
D
SAM:SAH ratio
P=1.00×10^-6
P=3.05×10^-5
P=0.0002
E
HCC70 xenograft
Average chow intake (g/mouse/day)
NS
F
Body weight (g)
Days post inoculation
G
TUNEL-positive cells (%)
Heart
Liver
Kidney
H
PDX4
I
J
K
BT549 xenograft
L
M

Supplemental Figure 10. Triple combination of MR and *MTAP* deficiency/inhibition combining PARPi is well tolerant. (**A**-**D**) Tumor growth inhibition (**A**), Kaplan-Meier survival curves (**B**), intratumor SAM (**C**), and SAM:SAH ratio (**D**) of BT549 xenograft tumors treated with olaparib, MTAPi, MR, or the indicated combination. All drugs were used at 10 mg/kg, intraperitoneally, 5 times per week. The control diet contained 0.86% methionine and methionine restriction diet contained 0.12% methionine. (**E**-**M**) Average chow intake, body weight, and TUNEL staining of heart, liver, and kidney of HCC70 (**E**-**G**), PDX4 (**H**-**J**), and BT549 (**K**-**M**) xenograft tumors with the indicated treatment. (**A** and **C**-**M**) Data are shown as the mean ± SD from one representative experiment of 5 mice per group, $n = 10$ mice per group in **B**. P values are indicated. Significance determined using (**A**, **C**-**E**, **G**, **H**, **J**, **K**, and **M**) 1-way ANOVA, (**B**) log-rank (Mantel-Cox), or (**F**, **I**, and **L**) 2-way ANOVA test.

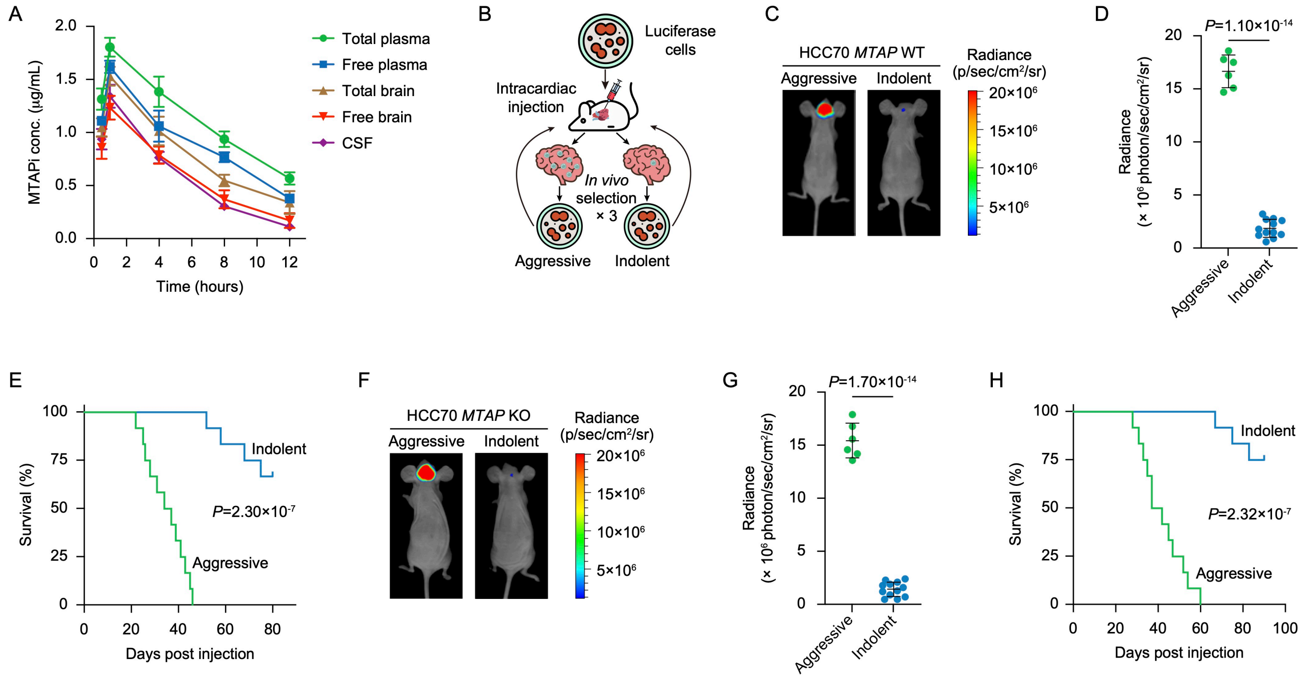

Supplemental Figure 11. Establishment of aggressive BrM HCC70 cells with WT or deleted *MTAP*. (**A**) *In vivo* pharmacokinetic study of MTAPi. (**B**) Schematic representation of *in vivo* selection for aggressive brain metastatic HCC70 cells with WT or deleted *MTAP*. (**C**-**H**) Representative *in vivo* bioluminescence imaging and quantification of radiance at day 35 post injection, and Kaplan-Meier survival curves of HCC70 aggressive or indolent BrM intracardiac injection mouse models with WT (**C**-**E**) or deleted *MTAP* (**F**-**H**). (**A**) Data are shown as the mean ± SD from 3 independent experiments, (**D** and **G**) data are shown as the mean ± SD from one representative experiment, n = 12 mice per group in **E** and **H**. P values are indicated. Significance determined using (**D** and **G**) unpaired t, or (**E** and **H**) log-rank (Mantel-Cox) test.
