# Supplementary material for: Targeting MTAP increases PARP inhibitor susceptibility in triple-negative breast cancer through a feed-forward loop
Source: J Clin Invest. 2025 Jul 1;135(13):e188120. doi: 10.1172/JCI188120 (PMC12208554; doi:10.1172/JCI188120)

Figure 1

Figure 1G

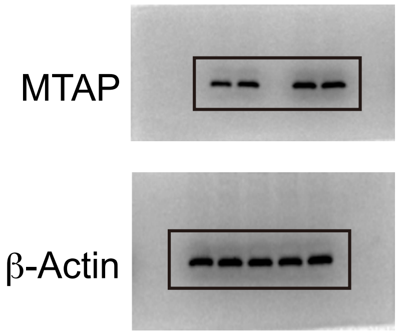

Figure 3

Figure 3A

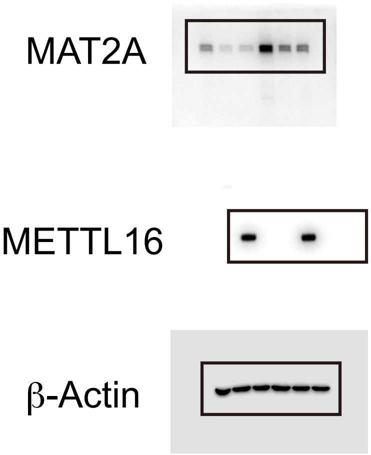

Figure 3E

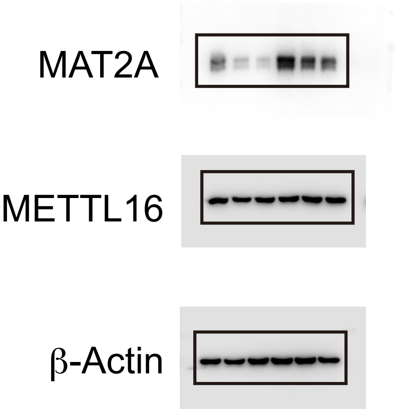

Figure 3G

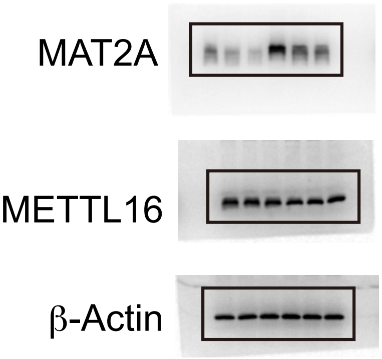

Figure 3J

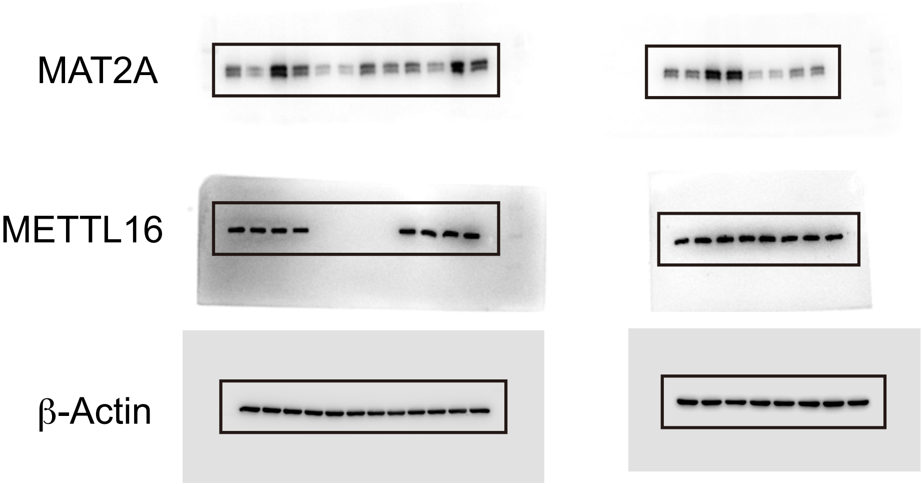

Figure 5

Figure 5A

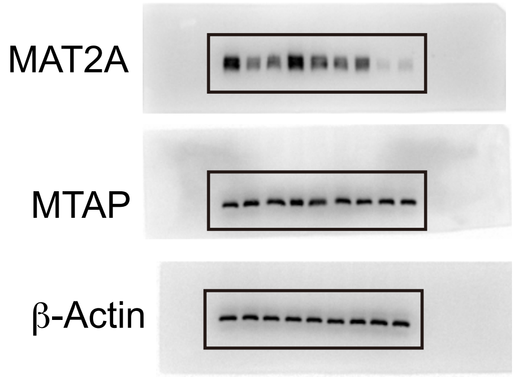

Figure 7

Figure 7I

RAD51
RPA32
MRE11
GAPDH
H3
MTAP

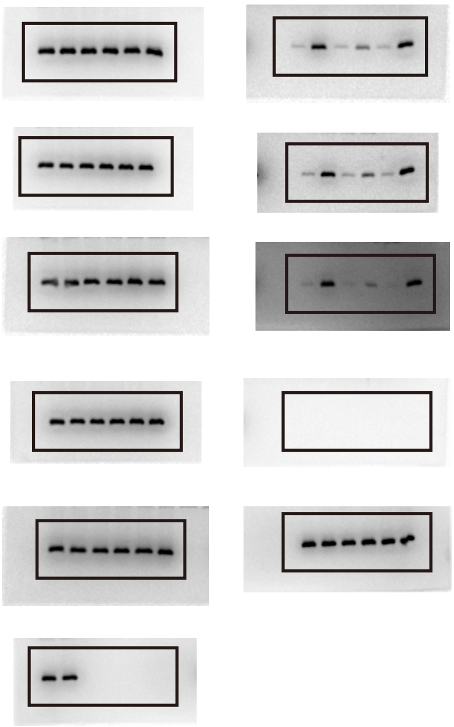

Figure 7J

ADMA
MRE11
MRE11
LaminB1

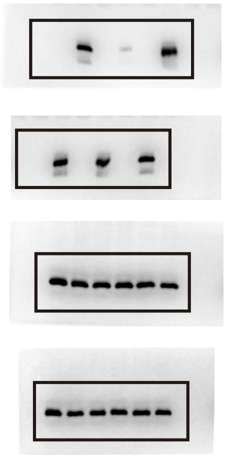

Figure 8

Figure 8A

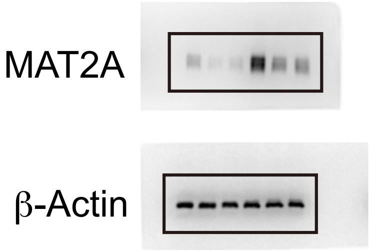

Supplemental Figure 1

Supplemental Figure 1A

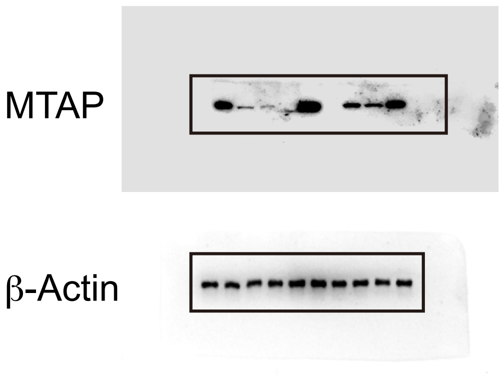

Supplemental Figure 1C

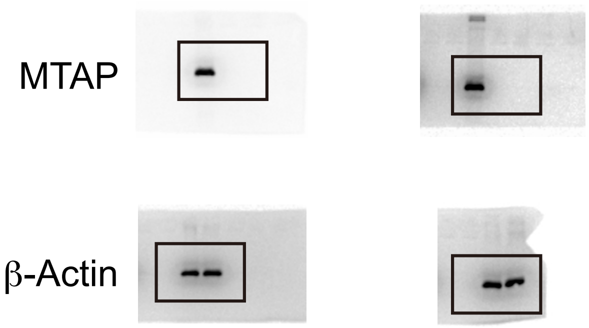

Supplemental Figure 2

Supplemental Figure 2G

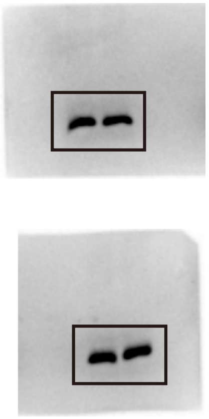

Supplemental Figure 3

Supplemental Figure 3A

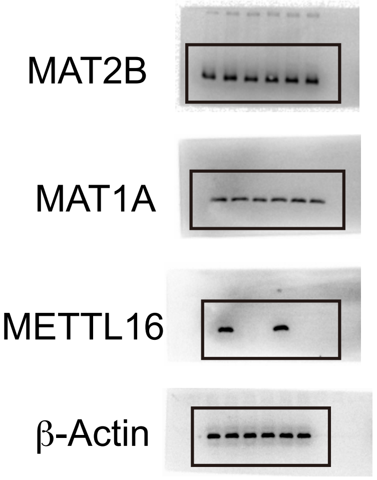

Supplemental Figure 3C

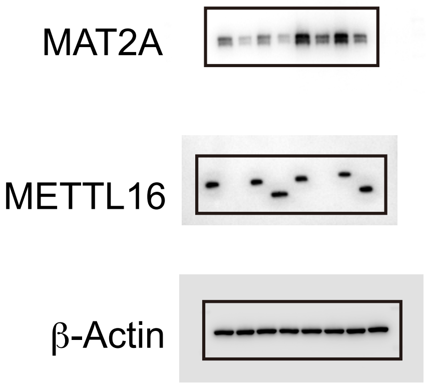

Supplemental Figure 3E

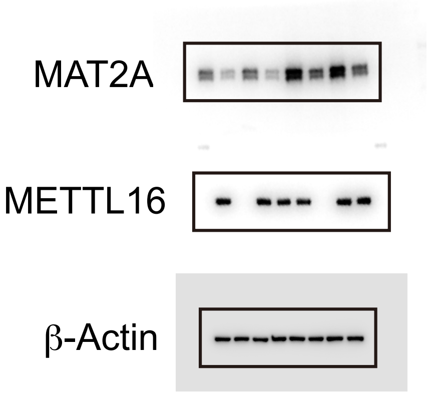

Supplemental Figure 3G

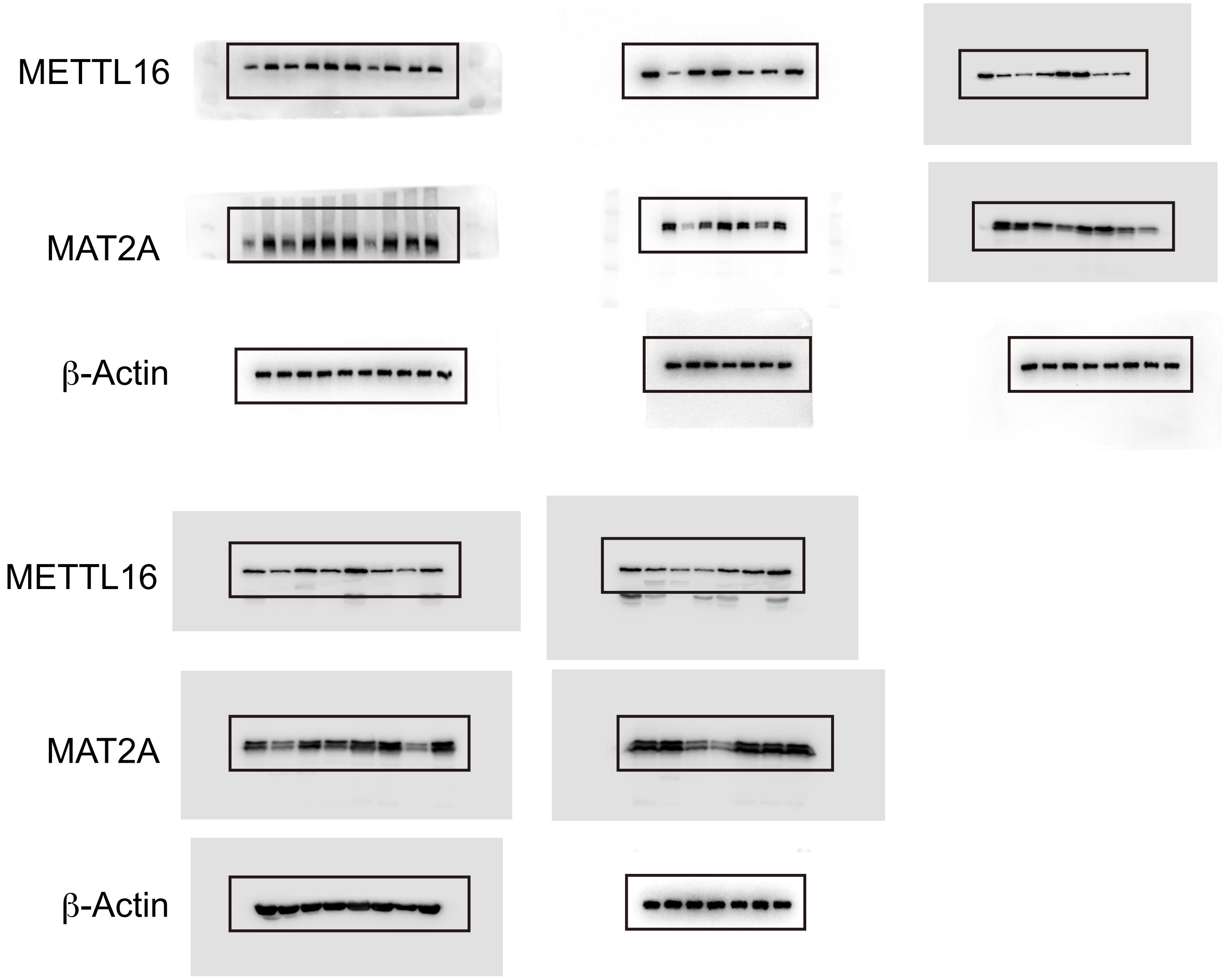

Supplemental Figure 4

Supplemental Figure 4A

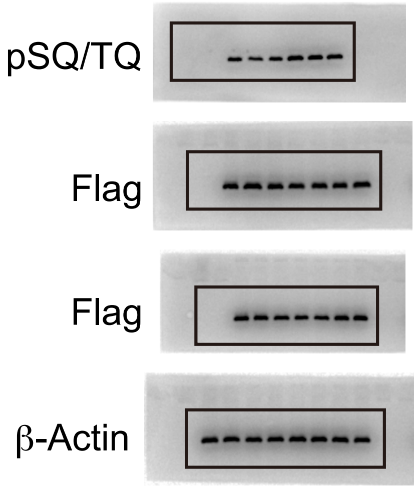

Supplemental Figure 4B

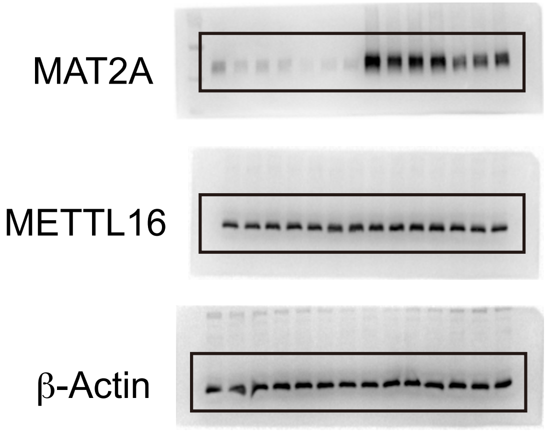

Supplemental Figure 4D

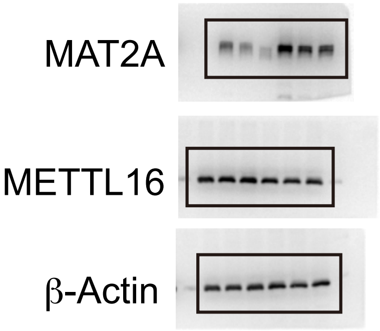

Supplemental Figure 4F

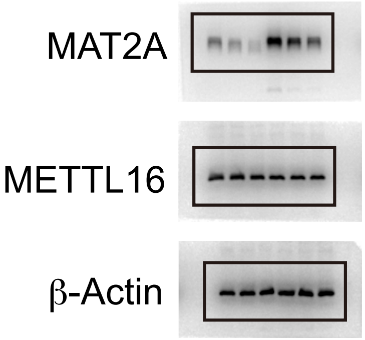

Supplemental Figure 4I

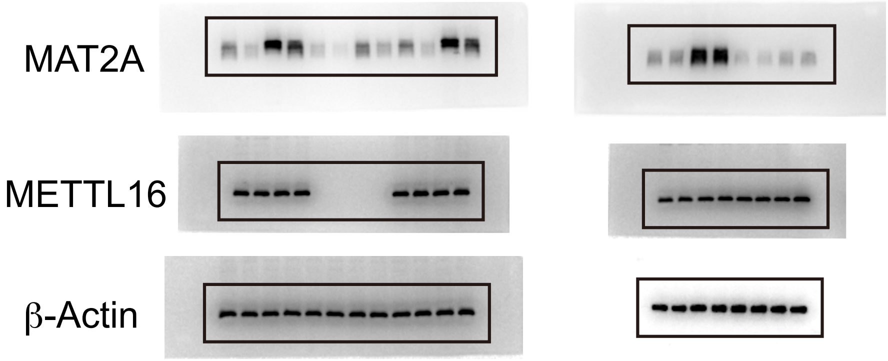

Supplemental Figure 5

Supplemental Figure 5A

MAT2A

METTL16

β-Actin

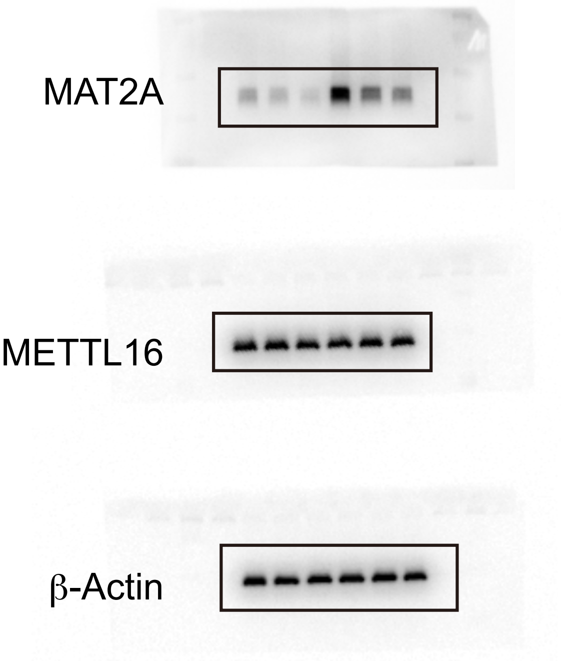

Supplemental Figure 5C

MAT2A

METTL16

β-Actin

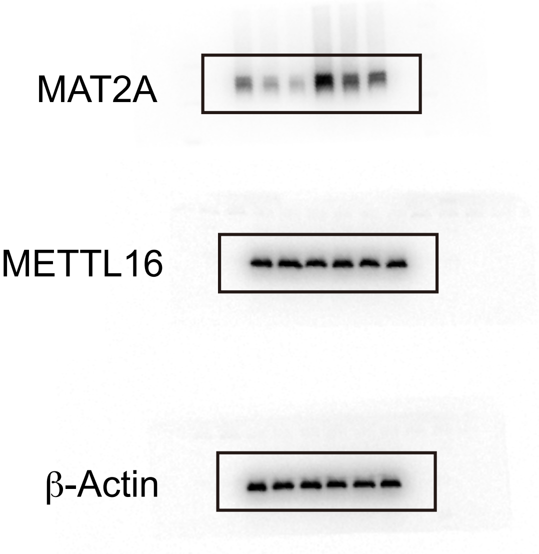

Supplemental Figure 5F

MAT2A

METTL16

β-Actin

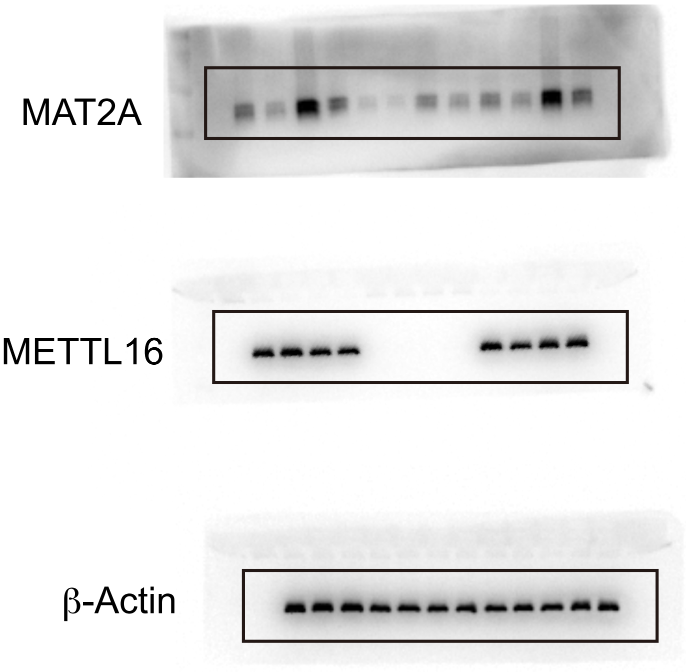

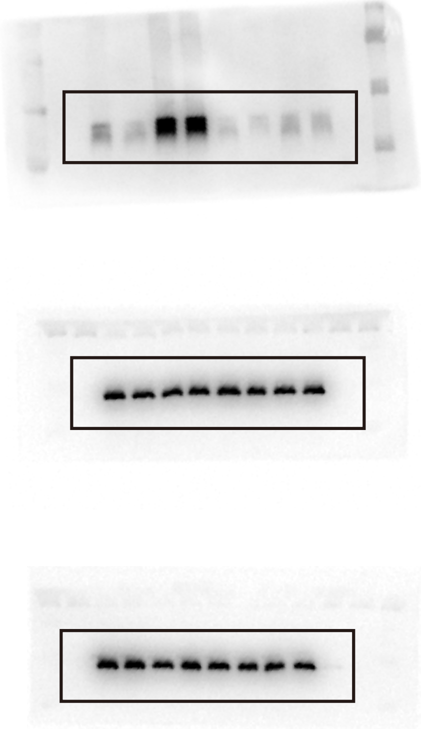

Supplemental Figure 7

Supplemental Figure 7A

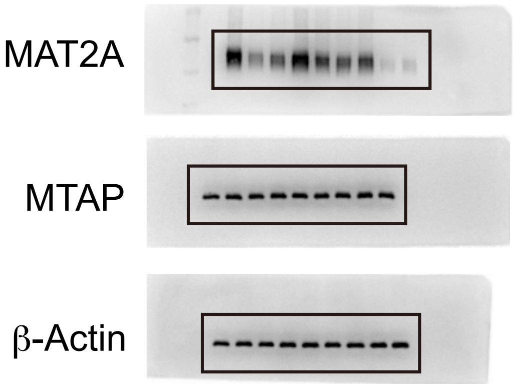

Supplemental Figure 8

Supplemental Figure 8I

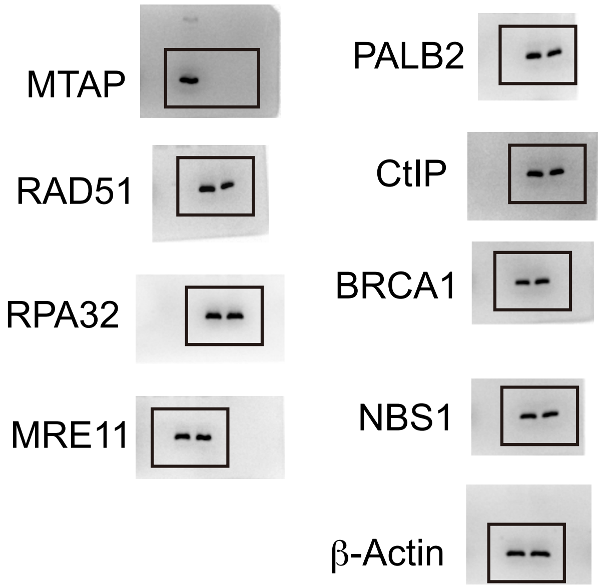

Supplemental Figure 8J

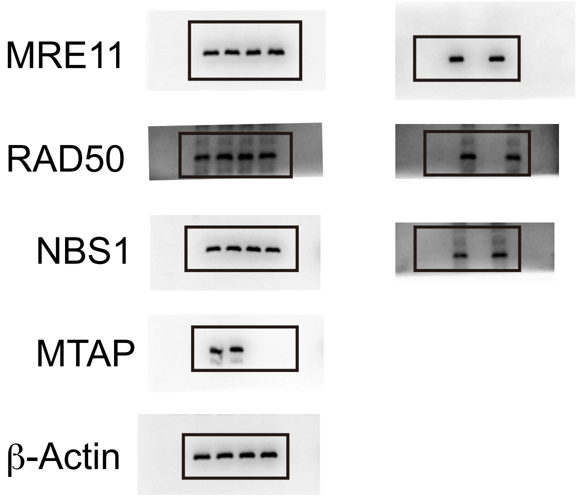

Supplemental Figure 8K

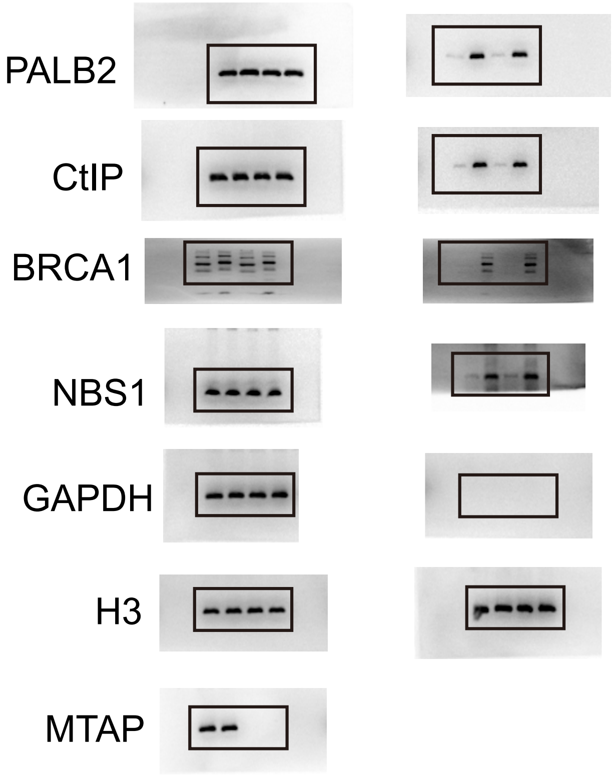

Supplemental Figure 9

Supplemental Figure 9A

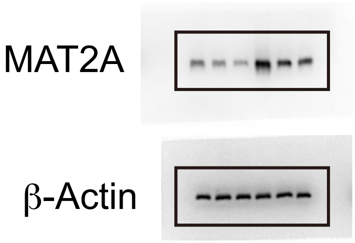

Supplement: Unedited blot and gel images [file jci-135-188120-s030.pdf]
